# Disruption of the *Snf1* Gene Enhances Cell Growth and Reduces the Metabolic Burden in Cellulase-Expressing and Lipid-Accumulating *Yarrowia lipolytica*

**DOI:** 10.3389/fmicb.2021.757741

**Published:** 2021-12-23

**Authors:** Hui Wei, Wei Wang, Eric P. Knoshaug, Xiaowen Chen, Stefanie Van Wychen, Yannick J. Bomble, Michael E. Himmel, Min Zhang

**Affiliations:** ^1^Biosciences Center, National Renewable Energy Laboratory, Golden, CO, United States; ^2^National Bioenergy Center, National Renewable Energy Laboratory, Golden, CO, United States

**Keywords:** *Yarrowia lipolytica*, cellobiohydrolase I, endoglucanase II, lipid metabolism, ATP citrate lyase, diacylglycerol acyltransferase, sucrose non-fermenting 1 gene, *Snf1* deletion

## Abstract

*Yarrowia lipolytica* is known to be capable of metabolizing glucose and accumulating lipids intracellularly; however, it lacks the cellulolytic enzymes needed to break down cellulosic biomass directly. To develop *Y. lipolytica* as a consolidated bioprocessing (CBP) microorganism, we previously expressed the heterologous CBH I, CBH II, and EG II cellulase enzymes both individually and collectively in this microorganism. We concluded that the coexpression of these cellulases resulted in a metabolic drain on the host cells leading to reduced cell growth and lipid accumulation. The current study aims to build a new cellulase coexpressing platform to overcome these hinderances by (1) knocking out the sucrose non-fermenting 1 (*Snf1*) gene that represses the energetically expensive lipid and protein biosynthesis processes, and (2) knocking in the cellulase cassette fused with the recyclable selection marker *URA3* gene in the background of a lipid-accumulating *Y. lipolytica* strain overexpressing ATP citrate lyase (*ACL*) and diacylglycerol acyltransferase 1 (*DGA1*) genes. We have achieved a homologous recombination insertion rate of 58% for integrating the cellulases-*URA3* construct at the disrupted *Snf1* site in the genome of host cells. Importantly, we observed that the disruption of the *Snf1* gene promoted cell growth and lipid accumulation and lowered the cellular saturated fatty acid level and the saturated to unsaturated fatty acid ratio significantly in the transformant YL163t that coexpresses cellulases. The result suggests a lower endoplasmic reticulum stress in YL163t, in comparison with its parent strain Po1g ACL-DGA1. Furthermore, transformant YL163t increased *in vitro* cellulolytic activity by 30%, whereas the “total *in vivo* newly formed FAME (fatty acid methyl esters)” increased by 16% in comparison with a random integrative cellulase-expressing *Y. lipolytica* mutant in the same YNB-Avicel medium. The *Snf1* disruption platform demonstrated in this study provides a potent tool for the further development of *Y. lipolytica* as a robust host for the expression of cellulases and other commercially important proteins.

## Introduction

*Yarrowia lipolytica* has a six-decade-long history of industrial application for the production of nutritional products (e.g., including omega-3 and omega-6 fatty acids, and carotenoids), organic acids (especial citric acid), and erythritol (see recent reviews Sitepu et al., 2014; Madzak, 2015; Zhu and Jackson, 2015). The first report of *Y. lipolytica* transformation (Davidow et al., 1985) was based on a lithium acetate method originally developed for *Saccharomyces cerevisiae* (Ito et al., 1983). Recently, *Y. lipolytica* has emerged as a promising oleaginous yeast for the development of engineered strains that produce biofuel-relevant products, such as lipids and fatty alcohols (Beopoulos et al., 2009; Tai and Stephanopoulos, 2013; Blazeck et al., 2014; Madzak, 2015; Wang et al., 2016). *Y. lipolytica* is a safe and robust microorganism that can grow on a broad range of substrates that include glucose, glycerol, and animal fat/crude oil waste. *Yarrowia* has also been engineered for xylose utilization to produce lipids (Ledesma-Amaro et al., 2016; Li and Alper, 2016; Markham et al., 2016; Rodriguez et al., 2016).

One hinderance for fulfilling the full potential of these natural *Yarrowia* strains is that they cannot utilize cellulosic biomass as a carbon source, which contributes to the relatively high cost for biofuel production. To overcome this hurdle, consolidated bioprocessing (CBP), in which microorganisms are engineered for the direct conversion of cellulosic biomass to biofuels and biochemicals has been proposed using *Clostridium thermocellum* (Lynd et al., 2005; Maki et al., 2009), *Caldicellulosiruptor bescii* (Chung et al., 2014), *S. cerevisiae* (Fan et al., 2012; Olson et al., 2012; Sun et al., 2012; Yamada et al., 2013; Kricka et al., 2014), *Kluyveromyces marxianus* (Chang et al., 2013), and *Myceliophthora thermophila* (Li et al., 2020). More recently, we and other workers have explored the potential of *Y. lipolytica* to serve as a CBP organism by screening and expressing fungal biomass-degrading enzymes in this normally non-cellulolytic host. These enzymes include **(1)** CBH I, CBH II, and EG II from *Trichoderma reesei* (Park et al., 2000; Boonvitthya et al., 2013; Wei et al., 2014), **(2)** a chimeric CBH I engineered from *T. reesei* and *Talaromyces emersonii* (Wei et al., 2014), and **(3)** XynII from *T. harzianum* and XlnD from *Aspergillus niger* (Wang et al., 2014a). The coculture of transformants expressing different individual CBH I, CBH II, and EG II enzymes by our group demonstrated synergy between these transformants (Wei et al., 2014). However, the coexpression of CBH I, CBH II, and EG II study was conducted using random insertion of these genes into the *Y. lipolytica* genome using a one-time use transformation selection marker, which restricts further engineering efforts. Additionally, we found that cellulase coexpression was likely a metabolic drain on the host cells, which reduced both the rate of cell growth and lipid accumulation, although this metabolic drain can be ameliorated by supplementation with a chemical chaperone, although at an additional economical cost.

We have now determined that these initial engineered strains may be improved by further genetic modification. Homologous recombination is a useful genetic process that allows the knockout of target genes and the knocking-in of specific gene and/or selection marker genes at the same time. Despite the initial unsuccessful attempts to disrupt the *Snf1* gene (sucrose non-fermenting 1) by double homologous recombination (Cervantes-Chávez and Ruiz-Herrera, 2007), some recent work successfully replaced targeted genes in *Yarrowia* (De Pourcq et al., 2012). As a recyclable selection marker, *URA3* has often been used for homologous recombination. Here, the target gene was replaced (i.e., disrupted) with the *URA3* selection marker cassette in *Y. lipolytica* (Callewaert et al., 2011). Moreover, the HisG*-URA3-*HisG recyclable cassette has been used for gene disruption in yeast more recently (Cauwood et al., 2013; Tian et al., 2013).

The ultimate goal of our studies is to develop a direct microbial sugar conversion (DMSC) platform for producing lipids, drop-in fuels, or chemicals from cellulosic biomass substrate. It is reported that SNF1, an AMP-activated serine threonine protein kinase, plays a primarily role in repressing the energetically expensive biosynthetic processes producing lipid and proteins in *S. cerevisiae* and mammalian cells (Hardie, 2007; Zhang et al., 2011). Moreover, knockout of the *Snf1* gene has been shown to increase lipid production in a naturally oleaginous strain of *S. cerevisiae* (Nicastro et al., 2015; He et al., 2018; Knoshaug et al., 2018). Given the unique role of *Snf1*, we can hypothesize that a disruption of *Snf1* in *Y. lipolytica* will loosen its negative signaling control over lipid and protein syntheses. This would result in the rewiring of the cellular metabolism and achieving increased lipid accumulation *via* increased protein production and secretion of cellulases without compromising the cell growth needed for DMSC. Thus, an important aspect of this work is aiming to improve the DMSC by improving the protein production and secretion of cellulases, especially the CBH and EG.

To test the above hypothesis, we built a series of constructs to first generate a high lipid-accumulating *Y. lipolytica* strain by overexpressing ATP citrate lyase (ACL) and diacylglycerol acyltransferase 1 (DGA1), and then engineered it further by homologous recombination to knockout the endogenous *Snf1* gene. A multiple cellulase expression cassette was then introduced into the cells. The *Snf1* disruption platform demonstrated herein can be applied to the further metabolic engineering of *Y. lipolytica* as a robust host to produce commercially important proteins and compounds, as is demonstrated here by lipid production from lignocellulosic biomass sugars.

## Materials and Methods

### Microorganisms and Maintenance Medium

It is noteworthy that the original parent strain for Po1g is W29, an industrial strain used to produce citric and isocitric acids (Jwanny et al., 1974; Maldonado and Charpentier, 1976; Fabre et al., 1990). A series of genetic manipulations had been conducted on W29, which made the Po1g a suitable host for expressing heterologous proteins as it lacks extracellular proteinases (Madzak et al., 2000). The *Y. lipolytica* strains used in this study are listed in Table 1. *Y. lipolytica* Po1g and vectors pYLEX1 and pYLSC1 were purchased from Yeastern Biotech Co. (Taipei, Taiwan). The strains were maintained at 28°C in the regular yeast peptone dextrose (YPD) agar, which contains 10 g L^–1^ of yeast extract, 20 g L^–1^ of peptone, 20 g L^–1^ of dextrose, and 15 g L^–1^ of agar.

**TABLE 1:** *Yarrowia lipolytica* strains and plasmids.

Strains or plasmids	Strain genotype and phenotype/plasmid components	Source
**Parent and derivative strains**		
Po1g	Genotype: *MatA, leu2-270, ura3-302*::URA3, *xpr2-332, axp-2*. Phenotype: Leu^–^, ΔAEP, ΔAXP, Suc^+^, pBR platform	Madzak et al., 2000
Po1g URA3^–^	Genotype: *MatA, leu2-270, xpr2-332, axp-2, ura3-, xpr2-332, axp-2*. Phenotype: Leu^–^, URA3^–^, ΔAEP, ΔAXP, Suc^+^, pBR platform	This study
Po1g ACL	Derivative of strain Po1g URA3^–^ by transforming with NotI-linearized plasmid pYLEX1-ACL for expressing *ACL* gene; URA3^–^	This study
Po1g DGA1	Derivative of strain Po1g URA3^–^ by transforming with NotI-linearized plasmid pMT015-YTEFin-DGA1 for expressing *DGA1* gene; URA3^–^	This study
Po1g ACL-DGA1	Derivative of strain Po1g URA3^–^ by transforming with NotI-linearized plasmid pMT015-YTEFin-DGA1-ACL for co-expressing *ACL* and *DGA1* genes; URA3^–^	This study
YL163-5 YL163-7	Derivative of strain Po1g-ACL-DGA1 by transforming with AscI/FseI-digested pNREL163; random insertion of Snf1up-*cbh1-cbh2-eg2*-HisG-*URA3*-HisG-Snf1down construct into host genome; URA3^+^	This study
YL163t	Derivative of strain Po1g-ACL-DGA1 by transforming with AscI/FseI-digested pNREL163; target insertion of Snf1up *cbh1-cbh2-eg2*-HisG-*URA3*-HisG-Snf1down construct into the disrupted *Snf1* gene site of host genome; URA3^+^	This study
YL165-1	Genotype: Po1f Δ*pex10*Δ*mfe1 leu^–^ URA^+^ DGA1 cbh1 cbh2 eg2* Phenotype: prevent peroxisome biogenesis and β-oxidation by Δ*pex10* and Δ*mfe1*, respectively; enhance lipid synthesis by *DGA1* overexpression; coexpress *cbh1*-*cbh2*–*eg2* genes	Wei et al., 2019
**Plasmids, constructs, and the cloned genes**		
pYLSC1 (i.e., pINA1296)	hybrid promoter hp4d; secretion signal (XPR2 pre region); selection marker gene *LEU2*; used as plasmid backbone for secretory protein expression	Madzak et al., 2000
pYLEX1 (i.e., pINA1269)	hybrid promoter (hp4d); selection marker gene (*LEU2*); used as plasmid backbone for intracellular protein expression	Madzak et al., 2000
pYLEX1-ACL	*M. musculus* ACL gene with promoter *p*EXP1 and terminator *t*EXP1; cloned in vector pYLEX1; Supplementary Figure 1	This study
pMT015-YTEFin	Constructed by replacing the hp4d promoter of pYLEX1 with the translation elongation factor-1a (TEF) promoter; used as plasmid backbone for intracellular protein expression	Tai and Stephanopoulos, 2013
pMT015-YTEFin-DGA1	*Y. lipolytica* DGA1 with TEFin promoter; cloned in vector pMT015-YTEFin; Supplementary Figure 1	This study
pMT015-TEFin-DGA1-ACL	pEXP1-*ACL*-tEXP1-pTEFin-*DGA1*; ACL gene with promoter *p*EXP1 and terminator *t*EXP1; DGA1 with TEFin promoter Supplementary Figure 1	This study
pNREL150	HisG-*URA3*-HisG with BamHI and HindIII sites at 5′ and 3′ ends, respectively; cloned into pUC57 by EcoRV site; used for building plasmid pNREL161; Supplementary Figure 2	This study
pNREL161	Snf1up-HisG-*URA3*-HisG-Snf1down in vector pUC57-Simple by EcoRV; used for building plasmid pNREL163; Supplementary Figure 2	This study
pNREL162	Chimeric *cbh1-cbh2-eg2* cassette; cloned in vector pUC57 by SalI and EcoRV; used for building plasmid pNREL163; Supplementary Figure 2	Wei et al., 2019
pNREL163	Snf1up-*cbh1-cbh2-eg2*-HisG-*URA3*-HisG-Snf1down in the vector pUC57-Simple; used for transforming Po1g ACL-DGA1 strain to obtain mutants YL163-1 to YL163-73; Supplementary Figure 2	This study

*See “Abbreviation” section for the descriptions of genes and their components.*

### Plasmid Construction and Strain Manipulations

Details of plasmid construction and strain manipulations are presented in Supplementary Additional File 1. We also note that the nomenclature for major genes/markers and enzymes used in this study was in line with their respective literature*: cbh1-cbh2-eg2* genes encoding CBH I-CBH II-EG II proteins (Wei et al., 2019), *ACL* gene encoding ACL protein, *DGA1* gene (GenBank accession number: YALI0E32769g) encoding DGA1 protein (Oelkers et al., 2002), *Snf1* gene (GenBank accession number: YALI0D02101g) encoding SNF1 protein (Tang et al., 2020), and *URA3* gene encoding URA3 protein (Larroudé et al., 2018). In addition, the small italic letter “*p*” represents promoter (to distinguish from small upright letter “p” that represents plasmid), while “*t*” represents terminator (Larroudé et al., 2018).

A *URA3*^–^ derivative strain of Po1g was generated and used in this study for overexpression of the *ACL* and *DGA1* genes, which was further used for the knockout *Snf1* gene and the knock-in of cellulases genes (Table 1). To create a host strain amenable to multiple gene insertions and knockouts, the previously described strategy of using *URA3* as a recyclable selection marker was used (Cauwood et al., 2013; Tian et al., 2013). To knock out the *URA3* gene and create a uracil auxotrophy, approximately 1 × 10^7^ *Y. lipolytica* Po1g cells were plated onto YNB plates with 5-fluoroorotic acid (5-FOA), with a composition of the following components as (g L^–1^): YNB base with ammonium sulfate 6.7, URA dropout mix 1.92, glucose 20, leucine 0.1, 5-FOA 2, and agar 20. The inoculated plates were incubated at 30°C until resistant colonies are observed. After 6 days of incubation, approximately 300 colonies per plate were noted. Eight colonies were restreaked to the same media to confirm resistance, on which all eight colonies regrew. These colonies were then streaked to the following five media types to confirm knockout of the *URA3* gene; all media contained (g L^–1^): YNB base 6.7, glucose 20, agar 20, and leucine 0.1, and 50 mg L^–1^ of either: uracil, orotic acid, orotidine, or uridine. The same media was also made lacking uracil. The wildtype Po1g grew on all of these media, whereas all of the mutants were only able to grow on the media containing uracil or uridine confirming knockout of the ability to produce uracil, thus, confirming disruption in the *URA3* gene function. All eight mutants were then tested for stability by growth on YPD (complete media) liquid media at 30°C and 225 rpm for 2 days followed by plating of a dilution series on YNB + leucine plates: with or without uracil. Of these eight mutants, seven showed no revertant colonies after 5 days of incubation. Glycerol stocks were made of the mutants and used for further cloning.

### Transformation and Colony Selection

*Yarrowia lipolytica* host cells were transformed with either restriction enzyme-digested constructs or linearized plasmid DNA, as described for each strain generated in Table 1. As described previously (Wei et al., 2014, 2019), a typical transformation of *Y. lipolytica* with restriction enzyme-digested constructs or linearized plasmid DNA described above was conducted using *YLOS* One Step Transformation system included in the *YLEX* expression kit (Yeastern Biotech Co., Taipei, Taiwan). *Y. lipolytica* cells grown in YPD pH4 liquid broth were collected from culture at 16∼24 h in baffled shaker flasks. The collected cells were washed two times with sterile dd-H_2_O to remove YPD residues and centrifuged again at 3,000 rpm for 5 min, followed by resuspending the cells (∼7 × 10^7^ cells per tube) in tubes containing 100 μl of freshly prepared *YLOS* cocktail containing the YLOS buffer, carrier DNA, dithiothreitol solution, and restriction enzyme-digested constructs or linearized plasmid DNA. The tubes were incubated at 39°C for 1 h, followed by spreading the entire cocktail on YNB selection plates. For the selection of auxotrophic recombinants (URA^+^), the transformed cells were grown on solid YNB medium (containing 2% glucose w/v, 0.67% yeast nitrogen base w/o amino acids, and 1.5% agar) with supplement of leucine, incubated at 28°C for 2∼4 days until the colonies of transformants developed.

### Genomic DNA Extraction, Primer Design, and PCR

Details are provided in Supplementary Additional File 1.

### SDS-PAGE and Western Blotting Analyses

Note that as illustrated in Supplementary Figure 2, each of the individual *cbh1*, *cbh2*, and *eg2* genes had a preceding XPR2 (alkaline extracellular protease 2 pre-region) as the signal sequence, which made these cellulases secrete into the medium during the culturing process. The supernatants of the strains were collected from culture grown in YPD pH 4.0 medium when OD_600_ reached a value of 10. The supernatant samples were subjected to the SDS-PAGE and Western blot analyses following the procedure described in the literature (Wei et al., 2019). The loading amount for SDS-PAGE separation was described in the legend of related figures. The Western blot analysis was conducted using mono anti-CBH I, mono anti-CBH II, and poly anti-EG II antibodies, respectively; these antibodies were generated and provided by the research group of Michael Himmel and Steve Decker at NREL.

### Cellulosic Substrates

Wet glass bead dispersed Avicel was used in this study. Details were described in a recent literature (Wei et al., 2019). Briefly, this substrate was prepared by suspending 1% Avicel in 50 mM acetate buffer, pH 4.8 in capped Fisherbrand glass bottles containing sterile glass beads (0.7 mm in diameter), and by shaking overnight at 180 rpm, 25°C. The volume of 1% Avicel:glass beads was 5:1.

### Cell Growth and Lipid Accumulation by Transformants in Lipid-Production Medium

Seed cultures of *Y. lipolytica* transformants were first prepared in 5 ml of YPD broth using 125-ml flasks and incubated at 28°C, 220 rpm. After 24 h, 2.5 ml of seed culture was inoculated to 25 ml of lipid production medium in a 250-ml shake flask and incubated with shaking at 28°C, 220 rpm.

It was noteworthy that glucose is the primary source of energy for *Y. lipolytica*, and also plays an important role in Snf1 signaling pathway, in general, as demonstrated in *S. cerevisiae* (Kayikci and Nielsen, 2015; Lin, 2021) and likely also in *Y. lipolytica*. Thus, a proportion of this work was focused on the use of glucose as a carbohydrate source for evaluating the performance of *Snf1* knockout mutants generated in this study.

The medium used for lipid production contained (given as g L^–1^): yeast extract 1.0 (NH_4_)_2_SO_4_, 0.5 (corresponding to 0.137 g L^–1^ ammonium); KH_2_PO_4_, 7.0; Na_2_HPO_4_, 2.0; MgSO_4_⋅7H_2_O, 1.5; CaCl_2_⋅2H_2_O, 0.15; FeCl_3_⋅6H_2_O, 0.15; ZnSO_4_⋅7H_2_O, 0.002; FeSO_4_⋅5H_2_O, 0.005; CoCL_2_⋅6H_2_O, 0.002; and MnSO_4_⋅5H_2_O, 0.002. The carbon source was glucose (2% w/v, i.e., 20 g L^–1^). The initial pH value of the medium was pH 6.0. In addition, leucine and uracil were supplemented as needed to the medium at a concentration of 0.1 g L^–1^ based on published work showing that the complementing of leucine and uracil biosynthetic capacity as well as the supplement of leucine and uracil increased lipid accumulation and growth rate in engineered, lipid-accumulating *Y. lipolytica* cells (Blazeck et al., 2014). The C/N ratio (g/g) of this lipid-production medium is 33, calculated using the equation below:


$$\frac{C}{N}=\frac{G\,l\,u\,c\,o\,s\,e\,g*40\%}{Y\,e\,a\,s\,t\,e\,x\,t\,r\,a\,c\,t\,g*10\%+a\,m\,m\,o\,n\,i\,u\,m\,s\,u\,l\,f\,a\,t\,e\,g*21\%+l\,e\,u\,c\,i\,n\,e\,g*11\%+u\,r\,a\,c\,i\,l\,g*25\%}$$

based on the factors that glucose contains 40% C, whereas yeast extract contains approximately 10% N, (NH_4_)_2_SO_4_ 21% N, leucine 11% N, and uracil 25% N by weight.

For the analysis of lipid production, cultures were harvested at 5 days (corresponding to the late stage of the stationary phase for high cell density) unless otherwise stated and centrifuged at 4,000 rpm. The cell pellet was separated and analyzed after centrifugation. Flask experiments were run in triplicates. Data were shown as the average of the triplicates and error bars represent the standard deviation of the triplicates.

### Growth Curves and Validation

Growth curves were obtained by following a modified protocol for cell inoculation, growth conditions, and turbidity measurements (Franden et al., 2013; Wang et al., 2014b). The detailed procedure was described recently (Wei et al., 2019) for growing the cells in YPD pH 4.0 medium (i.e., a cellulase compatible production medium) at an initial OD_600_ of 0.25 and then distributing aliquots into Bioscreen C microplates (i.e., three wells per cell line; 300 μl per well). The Bioscreen C microplates were incubated and shaken at 30°C for 5 days, with absorbance read every 15 min. Turbidity was spectrophotometrically measured using a wide band filter (420–580 nm) and logged using EZ Experiment software. The turbidity data in spreadsheets were exported to Microsoft Excel, and the data were averaged from three replicates of cell samples.

### *In vitro* Enzyme Activity Assays for Crude Enzymes

The combinational enzymatic activity of coexpressed CBH I–CBH II–EG II in the supernatant of *Y. lipolytica* strains was measured using a published protocol (Wei et al., 2019). Briefly, the supernatants were collected from the YPD culture of CBH I–CBH II–EG II coexpressing transformants after 5 days of shaking at 30°C, 200 rpm. A 0.5 ml of the supernatant was incubated with 0.5 ml of 1% glass bead-dispersed Avicel at 50°C for 2 and 24 h. The released sugars were measured by HPLC. The conversion rate of “% Avicel to glucose” was calculated as the measured “total glucose equivalent released in g L^–1^” divided by 5 g L^–1^, which was the amount of total glucose equivalent released for theoretical 100% Avicel conversion.

### Cellulose Utilization by Transformants in YNB–Avicel Medium

The *in vivo* utilization of cellulose by transformants in YNB–Avicel medium was investigated, using the procedure described recently (Wei et al., 2019). Briefly, seed cell culture was grown in 20 ml of YPD medium in a 125-ml baffled flask, shaking overnight at 28°C, followed by centrifugation for cell collection. The sterile ddH_2_O-washed cells were used to inoculate 100 ml of mineral medium in 500-ml of baffled flasks with an initial OD of 1.6 (equivalent to approximately 1 g DCW L^–1^; dry cell weight basis). The mineral medium was supplemented with 2.7 g of Avicel (i.e., 2.7% w/v) as well as with β-glucosidase (*Aspergillus niger* BGL: cat. no. E-BGLUC, Megazyme, International Ireland Ltd., Wicklow, Ireland), at a concentration of 2 mg of BGL per gram of cellulose substrate (Selig et al., 2014; Wei et al., 2014; Xu et al., 2017). The flasks that contained the cell-medium–Avicel–BGL mixtures were incubated in a rotary shaker at 200 rpm and 28°C for 5 days, which was the same cell growth time length used for growing the cells in lipid production medium. The cells were centrifuged, freeze-dried, and subjected to Avicel residue analysis, as described in the following section. The experiments were run in three biological triplicates.

### Quantification of Avicel Residues and Cell Weight in Avicel–Yeast Cell Mixture

The determination of Avicel residues and cell dry weight was conducted using standard analytical procedures previously described (Wei et al., 2014, 2019; Guo et al., 2017).

### Fatty Acid Methyl Ester Analysis

The procedure for fatty acid methyl ester (FAME) analysis was described previously (Wei et al., 2013). Briefly, the cells were collected by centrifugation, freeze-dried, and subjected to FAME analysis using a one-step extraction and esterification method (Laurens et al., 2012). A mixture of 0.3 ml of HCL–methanol (5% v/v) and 0.2 ml of chloroform–methanol (2:1 v/v) was added to 7–11 mg of lyophilized cell pellets, vortexed in a sealed glass vial, and heated to 85^°^C for simultaneously solubilizing and transesterifing the lipids. The resultant fatty acid methyl esters (FAMEs) were extracted with hexane (1 ml) for 1 h at room temperature and analyzed by gas chromatography with flame-ionization detection (GC-FID) with an Agilent 6890N chromatograph equipped with an HP5-MS column (Agilent, United States). The cell mass samples were analyzed by using 250 μg of C^13^-FAME as a quantitative internal standard. FAME quantification was based on the integration of individual fatty acid peaks in the chromatograms; peak areas were compared with those for a five-point calibration curve of an even-carbon-chain FAME standard mixture of 14 individual fatty acids (C8–C24; catalog no. 18918, Sigma-Aldrich). Total FAME content was determined as the sum of the even-numbered FAMEs.

## Results and Discussion

### Expressing Heterologous ATP Citrate Lyase and Endogenous Diacylglycerol Acyltransferase 1 Genes in *Yarrowia lipolytica*

The initial step of strain engineering in this study was to generate *Yarrowia* mutants expressing the *ACL* and *DGA1* genes. ATP citrate lyase (ACL) is the main enzyme responsible for cleaving cytosolic citrate into acetyl-CoA and plays an important role in *de novo* lipid synthesis. ACL enzymes from fungi and yeast are composed of two different subunits (α_4_β_4_) (Nowrousian et al., 2000; Blazeck et al., 2014). Previously, either the ACL1 alone (Tai and Stephanopoulos, 2013) or both subunits (ACL1 and ACL2) of *Y. lipolytica* were overexpressed (Blazeck et al., 2014) in *Y. lipolytica*. In contrast, mammalian ACL is a single polypeptide homotetramer (α_4_) with a subunit of about 1,100 amino acids (Elshourbagy et al., 1990; Elshourbagy et al., 1992). It was reported that the overexpression of *Mus musculus* ACL enhanced the lipid accumulation in *Y. lipolytica* (Elshourbagy et al., 1992; Zhang et al., 2014), thus, mouse ACL (NM_001199296) was selected for overexpression in *Y. lipolytica* in this study because of the relative simplicity (only a sole gene was required for expression).

Diacylglycerol acyltransferase (DGA) is the final step of the triacylglycerol (TAG) biosynthesis pathway, as illustrated in Supplementary Figure 1. Overexpression of the *Y. lipolytica DGA1* gene (YALI0E32769g) increased lipid production in *Y. lipolytica* (Athenstaedt, 2011; Qiao et al., 2015); thus, it was selected here to be coexpressed with *M. musculus* ACL to boost lipid production in *Y. lipolytica*. Together, the coexpression of ACL and DGA1 serves as a push-and-pull strategy to enhance lipid accumulation in *Y. lipolytica*.

Plasmids pYLEX1-ACL, pMT015-YTEFin-DGA1, and pMT015-YTEFin-DGA1-ACL transformed into the Po1g URA3^–^ mutant at the pBR322 docking platform within the genome of the host strain. The resultant transformants were named as Po1g ACL, Po1g DGA1, and Po1g ACL-DGA1, respectively, as listed in Table 1.

### Lipid Production in Mutant Po1g ATP Citrate Lyase–Diacylglycerol Acyltransferase 1

**FIGURE 1:**
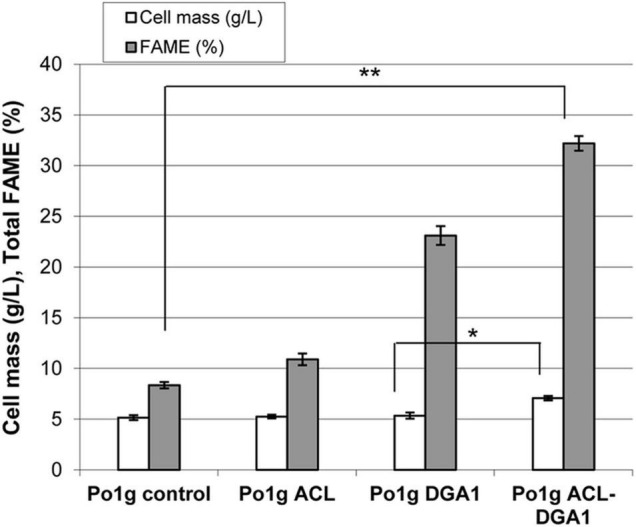
Cell mass and lipid production by *Yarrowia lipolytica* strains expressing ATP citrate lyase (ACL), diacylglycerol acyltransferase 1 (DGA1), and ACL-DGA1 after growth in lipid-production medium for 5 days. Data presented are the average of three replicate measurements ± SEM. Cell mass among the first three strains (Po1g control, Po1g ACL, and Po1g DGA1) are not significantly different, but are significantly different from that of the fourth strain Po1 ACL-DGA1 (*p* < 0.05, indicated by the *). In contrast, the values of % FAME among the four strains are significantly different (*p* < 0.01, indicated by the **) from each other.

Two lipid synthesis-related genes, *M. musculus ACL* and *Y. lipolytica DGA1*, were overexpressed in *Y. lipolytica* Po1g URA3^–^ (referred to as Po1g control strain in below sections). Mutant Po1g ACL-DGA1 accumulated lipids to a level of approximately 35% FAME after growth for 5 days in lipid production medium, which is approximately four times that of the parent control strain. Furthermore, the cell mass (in dry weight) of Po1g ACL-DGA1 was 37% higher than that of the parent control strain Po1g (7.1 vs. 5.2 g DW cell mass L^–1^) (Figure 1).

Since Po1g ACL-DGA1 showed significantly improved cell mass and lipid content compared with the Po1g control strain, the lipid composition profiling of these strains was compared. Po1g ACL-DGA1 cells shifted from unsaturated to saturated FA, and increased by approximately 50% for saturated C16:0 and 150% for saturated C18:0 over those of the Po1g control strain, respectively (Table 2, indicated by upward arrows); in contrast, Po1g ACL-DGA1 had decreased percentage of unsaturated C16:1n11, C18:1n7, C18:2n6, and C18:3n3 (Table 2, indicated by downward arrows). This resultant DGA1-ACL-overexpressing strain with increased lipid accumulation was used as the recipient strain for the coexpression of CBH I, CBH II, and EG II.

**TABLE 2:** Lipid composition in Po1g ATP citrate lyase (ACL)-diacylglycerol acyltransferase 1 (DGA1) cells.

(%)	Total FAME	C16:0	C16:1n11	C16:1n7	C17:0	C17:1	C18:0	C18:1n9	C18:1n7	C18:2n6	C18:3n3
Po1g control	7.9	11.0	2.3	5.9	0.2	0.1	4.5	51.1	1.6	17.1	1.3
Po1g ACL-DGA1	32.2 ↑	14.9 ↑	1.0 ↓	6.2	0.2	0	11.4 ↑	51.9	1.0 ↓	9.7 ↓	0 ↓

*These data represent an average of triplicates with standard deviation of <5%. The changes in lipid composition in Po1g ACL-DGA1 vs. Po1g control cells are indicated by upward or downward arrows, with statistical significance of p < 0.01 between the two strains.*

### Construct Building and Transformation for Knocking Out *Snf1* and Knocking in Cellulase Cassettes in Po1g ATP Citrate Lyase–Diacylglycerol Acyltransferase 1 Strain

The goal of this study was to develop a genetic system for integrating the *cbh1–cbh2–eg2* expression cassettes into the host strain at its *Snf1* gene site with the recyclable selection marker (*URA3*). Construct 163 was built by fusing Snf1up-HisG-*URA3*-HisG-Snf1down with *cbh1*-*cbh2*-*eg2*, using the steps illustrated in Supplementary Figure 2.

As listed in Table 1, the plasmid pNREL163, which harbors approximately the 14-kb construct of Snf1up–*cbh1–cbh2–eg2*–HisG–*URA3*–HisG–Snf1down, was digested with AscI/FseI, and transformed into the Po1g ACL-DGA1 strain generated earlier. Strains were then produced that incorporated the *cbh1–cbh2–eg2* cassettes and *URA3* gene into the genome of host cells using the procedure described in the “Materials and Methods” section. In total, 73 URA3^+^ transformants were obtained from YNB plates lacking uracil. The individual cell colonies were picked and restreaked on YNB plates lacking uracil and were further analyzed.

**FIGURE 2:**
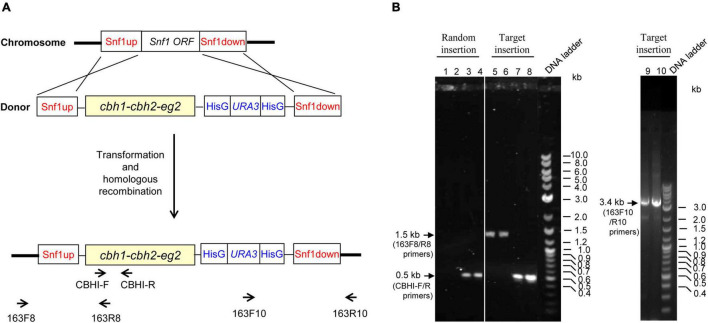
Primer design and PCR and agarose gel electrophoresis analyses for the insertion of Snf1up–*cbh1–cbh2–eg2*–HisG–*URA3*–HisG–Snf1 down construct (i.e., construct 163) into the genome of Po1g ACL-DGA1 strain *via* either random insertion or target insertion at the disrupted *Snf1* gene site. **(A)** Illustration of primer design are described in greater detail in Supplementary Table 1. **(B)** PCR characterization for the mode of integration into the host genome in representative mutants. The sizes for the PCR products with specific primers are labeled with arrows.

PCR analysis of transformants was conducted to distinguish homologous recombination from random insertion, using extracted genomic DNA from transformants as templates (see Figure 2A). The PCR band patterns for transformants with random vs. *Snf1* site insertions are illustrated in Figure 2B. Transformants with random insertion (represented by transformants #5 and #7, which were named as YL163-5 and YL163-7, respectively) were confirmed to be the mutants with construct 163 randomly inserted into the host genome; their PCR had no amplification using *Snf1*-flanking primers 163F8 and 163R8 (Figure 2B, lanes 1–2), but had amplification with an amplicon size of ∼0.5 kb using CBH I-F and CBH I-R primers aligning to the *cbh1* cassette region (Figure 2B, lanes 3–4). In contrast, transformants with *Snf1* site-specific insertions, in addition to the ∼0.5 kb band amplified from using CBH I-F and CBH I-R primers (Figure 2B, lanes 7–8) showed PCR bands detected with both Snf1up-flanking primers (163F8 and 163R8; ∼1.5 kb band; Figure 2B, lanes 5–6) and Snf1down-flanking primers (163F10 and 163R10; ∼3.4 kb band; Figure 2B, lanes 9–10).

Of the 73 transformants screened, 42 transformants showed that the engineered construct was inserted at the *Snf1* gene site of the genome with a homologous recombination insertion rate of 58%, illustrating that the cellulase cassettes were integrated at the desired locus. The representative strain carrying the target insertion of *cbh1–cbh2–eg2*–HisG–*URA3*–HisG at the *Snf1* site was named YL163t. It is noteworthy that our results were consistent with the finding in the literature, which showed that the integration into *Y. lipolytica* usually occurs without homologous recombination leading to random insertion (Hong et al., 2013). However, homologous recombination does occur with homologous sequences longer than 1 kb (Verbeke et al., 2013). In our study, the relatively high homologous recombination insertion rate (58%) can be attributed to the long homologous sequences (i.e., 1.2 kb) of Snf1up and Snf1down used in construct 163 to target the endogenous *Snf1* gene in the host genome.

### Expression of Cellobiohydrolase I, Cellobiohydrolase II, and Endoglucanase II in YL163t: Western Blot Analysis

**FIGURE 3:**
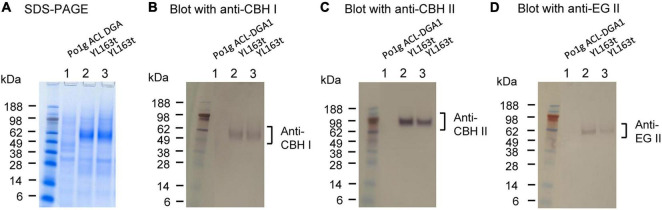
SDS-PAGE and Western blot analyses of the YL163t transformant coexpressing the cellulases cellobiohydrolase (CBH) I, CBH II, and endoglucanase (EG) II. After SDS-PAGE **(A)**, Western blot analyses were conducted using **(B)** anti-CBH I, **(C)** anti-CBH II, and **(D)** anti-EG II antibodies, respectively. The loading amount was 22.5 μl of supernatant for each well. SDS-PAGE was ran in NuPAGE MES buffer; SeeBlue Plus2 Prestained Protein Standard (LC5925; Invitrogen, NY, United States) was used as the markers.

The targeted insertion mutant YL163t expressing *cbh1–cbh2–eg2*–HisG–*URA3*–HisG at the disrupted *Snf1* gene site was used for cellulase expression analysis. YL163t and the parent control strain were cultured in flasks containing YPD pH 4.0 medium (as a cellulase production medium), shaken at 30°C. The supernatants of the cultures were collected when the OD_600_ value of the culture reached 10, and analyzed by SDS-PAGE and Western blotting, and the results are shown in Figure 3. Compared with the parent control strain Po1g ACL-DGA1, the YL163t transformant showed multiple proteins in the molecular size range between 51 and 64 kDa for CBH I, CBH II, and EG II (Figure 3A), and the Western blot analysis confirmed the expression of these proteins (Figures 3B–D).

### Effect of *Snf1* Disruption on Lipid Production in Cells Grown in Lipid Production Medium

**FIGURE 4:**
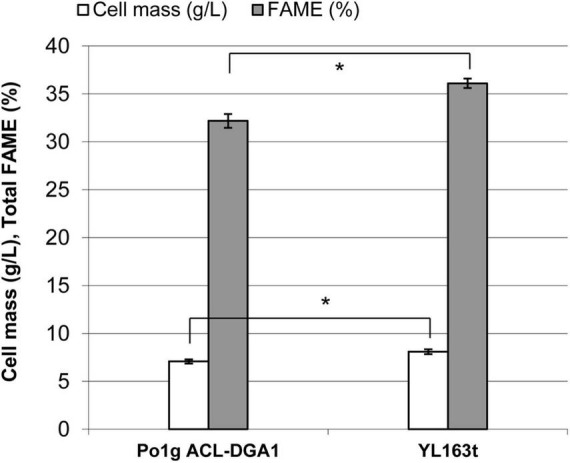
Cell mass and lipid production by mutant YL163t having an *Snf1* gene deletion and coexpression of *cbh1*, *cbh2*, and *eg2* genes, in comparison with its parent strain Po1g ACL-DGA1 after growth in lipid production medium for 5 days. Data presented are the average of three replicate measurements ± SEM. *Statistical significance of *p* < 0.05 between the two strains.

Lipid production by YL163t was evaluated using the cells cultured in the lipid production medium. After 5 days of culturing, the cell mass of YL163t was 8.1 g DCW L^–1^, a 14% increase (*p* < 0.05) over that of the parent control strain Po1g ACL-DGA1 (7.1 g DCW L^–1^) (Figure 4), which can be attributed to *Snf1* disruption in YL163t. Similarly, the % FAME on dry weight basis of YL163t was 36.5%, which constituted a 13% increase (*p* < 0.05) over that of the parent control strain Po1g ACL-DGA1 (32.2% FAME) (Figure 4). Our observation was consistent with the report that an *Snf1* deletion mutant of *Y. lipolytica* accumulated more fatty acids constitutively than the wild-type strain (Seip et al., 2013).

**FIGURE 5:**
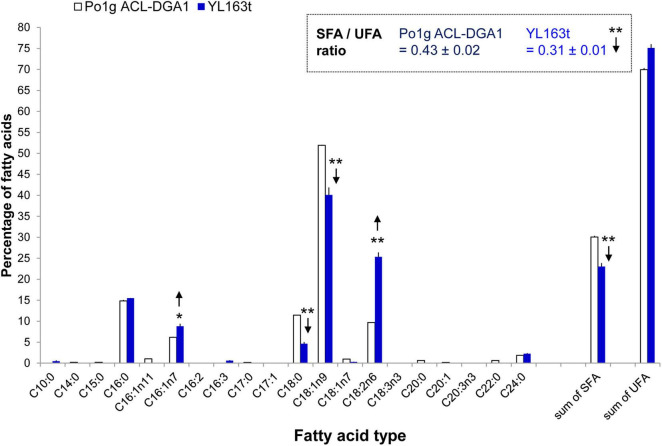
Fatty acid profiles of *Y. lipolytica* YL163t vs. the parent control strain Po1g ACL-DGA1 culture in medium. Data presented are the average of three replicate measurements ± SEM. * and **Statistical significance of *p* < 0.05 and *p* < 0.01, respectively, for comparing the fatty acid types between the two strains. SFA, saturated fatty acids; SFA/UFA, ratio of saturated fatty acids to unsaturated fatty acids; UFA, unsaturated fatty acids.

FAME analysis showed that the YL163t cells had significantly less saturated C16:0 and the sum of the saturated fatty acids (SFA), leading to a lower SFA/UFA ratio of 0.31, compared with the SFA/UFA ratio of the control strain Po1g ACL-DGA1 of 0.43, i.e., reduced the ratio by 27% (Figure 5, indicated by downward arrows). Because intracellular accumulation of SFAs is associated with ER stress (Pineau et al., 2009), our observation of lower SFA in YL163t suggests that the deletion of *Snf1* gene reduces ER stress in *Y. lipolytica* and likely makes cells more robust for metabolic functionality, in general. The suggested relationship between *Snf1* gene and ER stress in *Y. lipolytica* is supported by literature, which demonstrated that the deletion of *Snf1* gene in yeast *S. cerevisiae* caused an increased and resistance to ER stress (Mizuno et al., 2015).

### Impact of *Snf1* Deletion on Cell Growth Rate in Cellulase Production Medium

**FIGURE 6:**
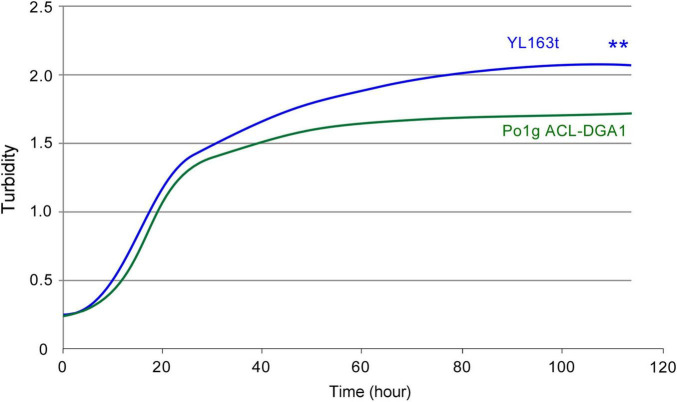
Bioscreen C growth curves of *Y. lipolytica* transformant YL163t that is coexpressing multiple cellulases and the parent strain. ** indicates statistical significance of *p* < 0.01 for comparing the two strains.

YL163t had a minimally shorter lag phase and grew slightly faster than its parent control strain during the very beginning of the exponential phase; it, thus, reached a higher turbidity throughout the plateau phase when grown in cellulase production medium (Figure 6). In both cellulase production medium (described here) and lipid production medium (as shown in Figure 4), *Snf1* deletion enhanced the cell growth. Our observation herein confirms a recent report that showed that *Snf1* disruption caused an increase in cell mass by 12% in HPDDS strain of *Y. lipolytica* (H222 Δ*POX1*-6 Δ*LEU2 + DGA1 DGA2* Δ*SNF1*), in comparison with *Snf1* unchanged HPDD strain (*H222 ΔPOX1-6 ΔLEU2 + DGA1 DGA2*) (Abghari and Chen, 2017).

### Hydrolytic Activities of Cellulases Secreted by YL163t

To measure the hydrolytic activities of the cellulases overexpressed by the mutants, supernatants were collected from their YPD pH 4.0 culture (i.e., using the cellulase-producing medium). The cellulase activities of the supernatants were measured by incubating the supernatant crude enzyme with the model substrate Avicel. As shown in Table 3, 0.02 g L^–1^ of cellobiose and 0.07 g L^–1^ of glucose were released after 1 h of saccharification with the supernatant of YL163t. These levels increased to 0.07 and 0.32 g L^–1^, respectively, after 24 h. This outcome represents an Avicel to glucose conversion rate of 7.8% after 24 h (Table 3). Comparatively, the supernatant of YL163t demonstrated a 30% higher Avicel conversion rate than that of the YL165-1 strain. This strain is a random integrative *Y. lipolytica* transformant for coexpressing the same multiple cellulase cassettes, which achieved an Avicel-to-glucose-equivalent conversion rate of 6.0% after 24 h of incubation under similar conditions (Wei et al., 2019).

**TABLE 3:** Enzyme activity of supernatant of YL163t coexpressing cellobiohydrolase (CBH) I–CBH II–endoglucanase (EG) II.

Enzyme-Avicel incubation time (50°C)	Strains	Reducing sugars released in saccharification mixture (g L^–1^)			
		Cellobiose	Glucose	Total glu equiv. released	% Avicel to glu equiv.
1 h	Po1g ACL-DGA1	0	0	0	0
	YL163t	0.02	0.07	0.09	1.8%
24 h	Po1g ACL-DGA1	0	0	0	0
	YL163t	0.07	0.32	0.39	7.8%

*Data presented were the average of three biological replicates, and the SEM (standard error of the mean) was <10%.*

*Glu equiv., glucose equivalent.*

It is noteworthy that in line with literature and the previous studies of our own group (Wei et al., 2014, 2019; Guo et al., 2017), the hydrolytic activity of cellulose-degrading enzymes was measured at 50°C (Table 3), which was higher than the growth temperature of 30°C for the culture of *Y. lipolytica* strains. Alternatively, the hydrolytic activity of cellulose-degrading enzymes can be also assayed under 30°C, which would require longer incubation time to achieve the same level of cellulosic substrate conversion, but should not affect the trend of the enzyme activities.

### *In vivo* Cellulose Utilization and Lipid Production by YL163t in YNB–Avicel Medium

The efficiency of the *Y. lipolytica* mutant YL163t in utilizing cellulose as the sole carbohydrate to produce lipids was examined by growing the transformant in mineral medium of YNB with Avicel and β-glucosidase as supplementation using the same procedure described in a recent study (Wei et al., 2019). The cells were harvested at 120 h, and the cell–Avicel mix residues were collected, freeze dried, and subjected to FAME and glucan content analyses.

The results showed that YL163t consumed 25.9% of the original Avicel content and produced 0.27 g DCW of cell biomass and 30 mg of FAME per g of Avicel consumed, leading to 11.5% in FAME value on DCW basis (Table 4). It is noteworthy that the “total newly formed FAME” of YL163t cells was 0.22 g L^–1^, 16% higher than that of the YL165-1 cells (i.e., 0.19 g L^–1^). The YL165-1 cells are the random integrative *Y. lipolytica* transformant for coexpressing the same multiple cellulase cassettes (Wei et al., 2019), when cultured and analyzed in parallel by the same researchers in our group. Thus, our analysis suggests that YL163t mutant has improved lipid production compared with the YL165-1 mutant grown in the YNB–Avicel medium.

**TABLE 4:** Cell mass and fatty acid methyl ester (FAME) of *Yarrowia lipolytica* transformant YL163t co-expressing CBH I, CBH II, and EG II and grown in YNB–Avicel medium.

Strain	Avicel consumed %^a^	Cell mass			FAME		
		Total DCW^b^	DCW of newly grown cells^c^	Yield^d^	Total newly formed FAME^e^	FAME %^f^	FAME yield
		g L^–1^	g L^–1^	g g^–1^ Avicel consumed	g L^–1^	DCW basis	mg g^–1^ Avicel consumed
YL163t	25.9 ± 0.5	2.9 ± 0.1	1.9 ± 0.1	0.27 ± 0.02	0.22 ± 0.03	11.5% ± 0.3%	32 ± 2

*^a^Initial concentration of Avicel in the medium was 27 g L^–1^; Avicel consumed % was measured as described in the “Materials and Methods” section.*

*^b^Total DCW was calculated as “dry weight of cell–Avicel pellet” - “amount of Avicel”.*

*^c^DCW of newly grown cells = total DCW - dry weight of initial inoculated cells (1 g L^–1^).*

*^d^Cell mass yield was calculated as “g dry weight of newly grown cells per g Avicel consumed”.*

*^e^“Total newly formed FAME” = “FAME of total cells” - “FAME of initial inoculated cells”.*

*^f^“FAME % DCW basis” = “total newly formed FAME”/“DCW of newly grown cells”. Data presented as the average of triplicate biological samples with SEM. DCW, dry cell weight.*

In addition, the “total newly formed FAME” value of YL163t cells (i.e., 0.22 g FAME L^–1^) can be subtracted from the “DCW of newly grown cells” (i.e., 1.9 g DCW L^–1^, Table 4), which led to 1.69 g L^–1^, a value representing new “purely grown” cell mass that excluded the FAME.

We note that the expression of the cellulases in YL163t mutant cells grown in the YNB-Avicel medium led to a lower lipid content (11.5% in FAME value on DCW basis, Table 4). Future studies should be conducted to investigate the profile of other products (including citric acid and other organic acids) produced by the cells, which can enable a carbon balance analysis that may help explain the observed lower lipid content in YL163t mutant cells grown in the YNB–Avicel medium.

## Conclusion

We successfully demonstrated the targeting of the *Snf1* site for coexpression of three cellulase genes (i.e., *cbh1*, *cbh2*, and *eg2*) fused with the recyclable selection markers HisG–*URA3*–HisG in *Y. lipolytica*. Of the 73 transformants obtained in this study, 42% of which were random insertion events, 58% were found to have the insertion of these genes into the host strain genome, as designed, at the target site of the *Snf1* gene, leading to the knockout of this gene through homologous recombination. Importantly, in the characterization of the *Snf1*Δ target inserted, cellulase-expressing transformant YL163t demonstrated that *Snf1*-deficient *Y. lipolytica* further increased the lipid accumulation compared with *Snf1*-positive control strains. *Snf1*Δ may have ameliorated the metabolic burden in heterologous cellulase-expressing *Y. lipolytica*, as reflected by **(1)** higher growth rate of YL163t, **(2)** higher *in vitro* cellulolytic activity, and **(3)** higher conversion rate of Avicel *in vivo* culturing of YL163t in minimal medium containing Avicel, compared with the parental control strain Po1 ACL-DGA1. Our results suggest that in the current model of SNF1 kinase-mediated crosstalk between lipogenesis and protein synthesis in yeast, the deletion of the *Snf1* gene affects this crosstalk in ACL-DGA1 overexpressing *Y. lipolytica* strains. The precise molecular mechanisms involved are worth further investigating, including a deeper investigation on the SNF1 signaling pathway in *Y. lipolytica*, which may lead to further increasing the strain fitness and production of heterologous enzymes.

It is also known that in addition to CBH and EG, active BGL is also needed for converting cellulose to glucose, primarily because of the inhibitory effect of cellobiose on the cellulases. Wild-type *Y. lipolytica* has a low level but, unfortunately, insufficient endogenous ability to digest cellobiose, as reported in literature (Kurtzman et al., 2011; Mirbagheri et al., 2012; Guo et al., 2015; Lane et al., 2015; Ryu et al., 2016) and discussed in our recent publication (Wei et al., 2019). Our studies have shown that the metabolic burden caused by the coexpression of CBH and EG enzymes in *Y. lipolytica* cells can be ameliorated either by adding a chemical chaperone into the culture medium (Wei et al., 2019) or by knocking out the Snf1 gene (as demonstrated in this study), thereby serving as a foundation for further strain development in the future. Future work will be focused on introducing the BGL genes into the CBH and EG coexpressing *Y. lipolytica* strains and upgrading them into fully cellulolytic, oleaginous strains. In addition, bench-scale bioreactor experiments will be performed on these upgraded engineering strains.

## Data Availability Statement

The original contributions presented in the study are included in the article/Supplementary Material, further inquiries can be directed to the corresponding authors.

## Author Contributions

MZ, MH, and HW led the project. YB coordinated the study. HW and WW conceived and designed the experiments. EK generated the Po1g Ura3^–^ strain. HW executed the DNA construct building, yeast transformation and mutant screening, SDS-PAGE, and Western blotting analysis. WW and XC conducted the cellulosic substrate preparation and lipid production and cellulase enzymatic activity analyses. SV conducted the FAME analysis. HW, WW, and EK prepared the initial draft of the manuscript. MH, YB, and MZ edited the manuscript. All authors read and approved the final manuscript.

## Author Disclaimer

The views expressed in this article are those of the authors and not necessarily those of the NHS, the NIHR, or the Department of Health and Social Care.

## Conflict of Interest

The authors declare that the research was conducted in the absence of any commercial or financial relationships that could be construed as a potential conflict of interest.

## Publisher’s Note

All claims expressed in this article are solely those of the authors and do not necessarily represent those of their affiliated organizations, or those of the publisher, the editors and the reviewers. Any product that may be evaluated in this article, or claim that may be made by its manufacturer, is not guaranteed or endorsed by the publisher.

## Abbreviations

ACL: ATP citrate lyase
BGL: β-glucosidase
CBH: cellobiohydrolase
DCW: dry cell weight
DGA: diacylglycerol acyltransferase
EG: endoglucanase
ER: endoplasmic reticulum
EXP1 promoter: promoter of *Y. lipolytica* export protein 1 gene
EXP1 terminator: terminator of *Y. lipolytica* export protein 1 gene
FAME: fatty acid methyl esters
GPD promoter: promoter of *Y. lipolytica* glyceraldehyde-3-phosphate dehydrogenase gene
hp4d: hybrid promoter derived from the XPR2 promoter
Lip2 terminator: terminator of *Y. lipolytica* lipase 2 gene
*p*: promoter
*Snf1* gene: sucrose non-fermenting 1
Snf1down: 1.2 kb upstream downstream nucleotide sequences of *Snf1* gene
SNF1up: 1.2 kb upstream nucleotide sequences of *Snf1* gene
*t*: terminator
TAG: triacylglycerol
Te: *Talaromyces emersonii*
TEFin promoter: TEF (translation elongation factor-1 α) intron promoter
Te-Tr: *Talaromyces emersonii–Trichoderma reesei*
Tr: *Trichoderma reesei*
XPR2: alkaline extracellular protease 2 pre-region (signal sequence)
YP: yeast extract-peptone (medium)
YPD: yeast extract–peptone–dextrose (medium).
