# Supplementary material for: Disruption of the Snf1 Gene Enhances Cell Growth and Reduces the Metabolic Burden in Cellulase-Expressing and Lipid-Accumulating Yarrowia lipolytica
Source: Front Microbiol. 2021 Dec 23;12:757741. doi: 10.3389/fmicb.2021.757741 (PMC8733397; doi:10.3389/fmicb.2021.757741)
Supplement: Supplementary file 1 [file Data_Sheet_1.pdf]

Manuscript title: Disruption of the *Snf1* gene enhances cell growth and reduces the metabolic burden in cellulase-expressing and lipid-accumulating *Yarrowia lipolytica*

Additional file 1:

File type: .pdf

List of contents: **Page no.**

Supplementary Materials and Methods

Outlines for plasmid construction and pathway engineering **2**

Plasmids for *ACL* and *DGA1* gene expression in *Yarrowia* **2**

Plasmids for HisG-*URA3*-His flanked by Snf1up and Snf1down, and fused with *cbh1-cbh2-eg2* **3**

Genomic DNA extraction, primer design, and PCR **4**

Supplementary Table S1. Sequences of forward (F) and reverse (R) primers **5**

Supplementary Figure S1. A three-step process for constructing plasmids pYLEX1-ACL, pMT015-TEFin-DGA1 and pMT015-TEFin-DGA1-ACL 6

Supplementary Figure S2. Main steps in building the constructs for *Snf1* depletion, recyclable *URA3* marker and cellulase expression in *Y. lipolytica* 7

Nucleotide sequences SEQ NO 1 to 11 **8**

Outlines for plasmid construction and pathway engineering

All plasmids used in this study are described in Table 1. For the deletion of *Snf1* and the expression of *cbh1*, *cbh2*, and *eg2* genes in *Yarrowia*, the overall approach for plasmid construction in this study can be divided into three parts, as described in below sections: **(1)** for *ACL1* and *DGA1* gene expression (**Supplementary Figure S1**), **(2)** for recyclable marker HisG-*URA3*-His flanked by *Snf1* gene's upstream (i.e. Snf1up) and downstream (i.e. Snf1down) sequences (**Supplementary Figure S2A**), and **(3)** for fusion with *cbh1-cbh2-eg2* cassette (**Supplementary Figure S2B**).

Plasmids for *ACL* and *DGA1* gene expression in *Yarrowia*

The constructs for expressing singular *ACL* and *DGA1* genes; as well as *ACL-DGA1*, were built in the backbone of vector pYLEX1 which contains an EXP1 promoter and EXP1 terminator for *ACL* gene, TEFin promoter and XPR2 terminator for *DGA1* gene, respectively, as outlined in Supplementary Figure S1.

Step 1. Plasmid pMT015-YTEFin-DGA1 (*i.e.,* construct 203) was constructed on the backbone of plasmid pMT015-YTEFin, which is a derivative of plasmid pYLEX1 with its hybrid promoter (hp4d) being replaced with *TEF*in (*i.e., TEF* promoter with intron). The map and sequence information were retrieved from the previously published supplemental materials in literature (Tai and Stephanopoulos, 2013), listed as SEQ NO 4 for the sequence record in this additional file 1. To insert *Y. lipolytica DGA1* gene (YALI0E32769g) into plasmid pMT015-*YTEF*in, we removed the ATG and added TAACCGCAG in the 5' end of the sequence of *Y. lipolytica* DGA1 gene to complete the intron after digestion with SnaBI. The synthesized *DGA1* sequence (1560 bp; this additional file 1, SEQ NO 5) had a blunt end on 5' and a NsiI site (ATGCAT) on the 3' end, and was cloned into the SnabI/NsiI double cut vector of pMT015-YTEFin. The resultant plasmid was named as pMT015-*YTEF*in-DGA1 (Supplementary Figure S1).

Step 2. Plasmid pY*LEX*1-ACL was constructed by a sequential cloning procedure described below. The related gene, promoter and terminator sequences were synthesized by GenScript with appropriate restriction sites at the 5' and 3' ends for assembling into destiny vector (Supplementary Figure S1), and described as below:

(1) Clone the synthesized promoter sequence of SalI-pEXP1-PmlI (*i.e.,* construct 205; 1014 bp, this additional file 1, SEQ NO 1) into vector pYLEX1 at the restriction cut sites of SalI-PmlI;

(2) Clone the synthesized terminator sequence of KpnI-tEXP1–SalI-ClaI (i.e. construct 206; 517 bp, this additional file 1, SEQ NO 2) into vector pYLEX1 at KpnI-ClaI cut sites;

(3) Clone the synthesized blunt end – *ACL*– stop codon KpnI (*i.e.,* construct 207; 3312 bp, this additional file 1, SEQ NO 3) into vector pYLEX1 at restriction cut sites of PmlI – KpnI. The resultant plasmid was named as pY*LEX1-ACL* (*i.e.,* construct 208, Supplementary Figure S1).

Step 3. To generate a single plasmid for co-expressing both ACL and DGA1, a promoter-gene-terminator cassette of pEXP1-*ACL-tEXP1* was cut from the above pYLEX1-*ACL* plasmid using restriction enzyme SalI and inserted into the SalI-cut pMT015-DGA1 with the same orientation. This resultant plasmid was named as pMT015-*TEFin-ACL-DGA1* (*i.e.,* construct 209, Supplementary Figure S1).

Plasmids for HisG-*URA3*-His flanked by Snf1up and Snf1down, and fused with *cbh1-cbh2-eg2*

The scheme for a six-step construct building process is illustrated in **Supplementary Figure S2**. The purpose of this process for building HisG-*URA3*-HisG cassette flanked by *Snf1* upstream (i.e. Snf1up) and downstream (i.e. Snf1down) sequences (Supplementary Figure S2A; steps 1-3), followed by fusing with *cbh1-cbh2-eg2* cassette (Supplementary Figure S2B; steps 4-6). The related genes and sequences were synthesized by GenScript with appropriate restriction sites at the 5' and 3' ends for cloning into destiny vectors as described in Table 1.

Steps 1 and 2. Synthesis of Snf1up and Snf1down

Y. lipolytica Snf1 gene's genomic sequence is 1.74kb region from base 236133 to 237872 (http://www.ncbi.nlm.nih.gov/nuccore/NC_006070.1?from=236133&to=237872&report=fasta). *Yarrowia Snf1* upstream 1.2 kb (base 234933 to 236132 of *Y. lipolytica* CLIB122 chromosome D complete sequence) and downstream 1.2 kb (base 237873 to 239073) sequences were retrieved from GenBank, and are also listed as SEQ NO 6 and NO 7, respectively, in this additional file 1. These sequences were synthesized with the appropriate restriction sites at the 5' and 3' end (**Supplementary Figure S2**).

Step 3. Synthesis of HisG-*URA3*-HisG

In HisG-*URA3*-HisG (i.e., construct 150, SEQ NO 8, this additional file 1), the *URA3* gene of *Y. lipolytica* flanked by direct repeats of *Salmonella typhimurium hisG* DNA (Supplementary Figure S2, Step 3). The 1157 bp fragment *HisG* gene of *S. typhimurium* was obtained from literature (Alani et al., 1987;Voth et al., 2001) and retrieved from GenBank (accession no. AF324729). The promoter, CDS and the terminator of *Y. lipolytica URA3* gene was retrieved from the genomic DNA sequence of U40564 at GenBank. The full length of U40564 is 1710 bp, among which 1-384 nt is promoter region, 385-1245 nt is the CDS of *URA3* gene (from start to stop codons), and 1246-1710 nt is the terminator region. We used 1-1545 nt (in which 1246-1545 nt was the core part of terminator) as the *URA3* cassette to be inserted between two HisG repeats.

Step 4. Assembly of Snf1up-HisG-*URA3*-HisG-Snf1down

For knocking out *Snf1* gene, the 1.2 kb DNA fragments of upstream and downstream nucleotide sequences of *Y. lipolytica Snf1* gene (as synthesized in Supplementary Figure S2, Steps 1 and 2) were added to the 5' and 3' end of HisG repeats, respectively (Supplementary Figure S2, Step 4). The assembled fragment was named as Snf1up-HisG-*URA3*-HisG-Snf1down *(i.e.,* construct 161, SEQ NO 9, this additional file 1), cloned in vector pUC57-Simple by EcoRV.

Step 5. *cbh1-cbh2-eg2* cassettes

cbh1-cbh2-eg2 cassettes *(i.e.,* construct 162, SEQ NO 10, this additional file 1) is a row of three cassettes of *cbh1-cbh2-eg2*, with SalI-PamlI site on its 5' end, and KpnI site on its 3' end. This construct was synthesized by our group in a recent study (Wei et al., 2019), in which the construct was built in the backbone of the vector pYLSC1 with the TEFin promoter and XPR2 terminator for Te-Tr (*Talaromyces emersonii-Trichoderma reesei*) chimeric *cbh1* gene, the GPD promoter and the Lip2 terminator for Tr *cbh2* gene, EXP1 promoter and EXP1 terminator for Tr *eg2* gene, respectively, as illustrated in Supplementary Figure S2.

Step 6. Construct of Snf1up-*cbh1-cbh2-eg2*-HisG-*URA3*-HisG-Snf1down

The construct of Snf1up-*cbh1-cbh2-eg2*-HisG-*URA3*-HisG-Snf1down (*i.e.,* construct 163, SEQ NO 11, 14064 bp, this additional file 1) was built by inserting the fragment of PamlI-*cbh1-cbh2-eg2*-KpnI (from construct 162) into the site of construct 161 between its Snf1up and first HisG, as illustrated in (Supplementary Figure S2).

Genomic DNA extraction, primer design, and PCR

Genomic DNA was isolated from *Y. lipolytica* cell pellets or colony patch by using the ZR Fungal/Bacterial DNA Miniprep kit (cat.# D6005; Zymo Research, Irvine, CA), and by following the procedure described in literature (Xu et al., 2017). The concentration of extracted genomic DNA was determined using Nanodrop, and adjusted to 20 ng μL^{-1} and stored at −80 ºC until use.

Primers were designed to characterize the modes for the insertion of construct 163 into the host cell genome. The initial screening primers were primers CBHI-F/CBHI-R that designed to align to *cbh1* cassette region of construct 163 and were used to confirm the insertion of construct 163 into the genome without distinguishing random insertion and target insertion. Furthermore, primers 163F8/163R8 and 163F10/163R10 were designed to flank the 5' end and 3' end of construct 163 presumably inserted into the genome at the site of disrupted *Snf1* gene in the desired mutants (see **Supplementary Table S1**, and as illustrated in the corresponding primer design and PCR result figure in the Results and Methods section).

To confirm the knocking-out of snf1 gene and knocking-in cellulase cassette in mutants. Their genomic DNA samples were PCR-amplified using the designed primers and the Q5 Hot start high-fidelity 2x master mix (New England BioLabs M0494). Based on the manual instruction for Q5 Hot start high-fidelity 2x master mix, thermal cycling conditions consisted of 30 s at 98 oC for initial denaturation, 32 cycles of amplification (10 s at 98 °C, 20 s at 60 °C, 30 s/kb at 72°C), 5 min at 72 ºC for final extension, and hold at 4 °C.

Supplementary Table S1. Sequences of forward (F) and reverse (R) primers. Primers were used to characterize the random verse target insertions of construct into the *Snf1* gene site in the genome of *Y. lipolytica* strains. An illustration of primer alignment regions can be found in Figure for PCR results in the Results and Discussion section.

Primer name	Sequences and primer alignment region	Use of PCR and amplicon size
CBHI-F<br>CBHI-R	F: TTCACCGCATCTAACCCACC;<br>align to the *cbh1* cassette region.<br>R: TCCACACCCCACAAAAAGAC;<br>align to the *cbh1* cassette region.	For initial confirming of construct 163 insertion into genome via either random or target insertion.<br>Product size: 517 bp.
163F8<br>163R8	F: TCGTCACCATGTCCTTCAGA;<br>flank 5' end of Snf1up.<br>R: CCCGCTACTGGGTCAATTT;<br>align to *cbh1* cassette.	For confirming construct 163's target insertion into genome at the site of disrupted *Snf1* gene in desired mutants.<br>Product size: 1493 bp.
163F10<br>163R10	F: TATCCGCATGATCTGTCCAA;<br>align to *URA3* nearing the second HisG.<br>R: GAGATCAAGCCGAAAAATGC;<br>flank 3' end of Snf1down.	For confirming construct 163's target insertion into genome at the site of disrupted *Snf1* gene in desired mutants.<br>Product size: 3354 bp.

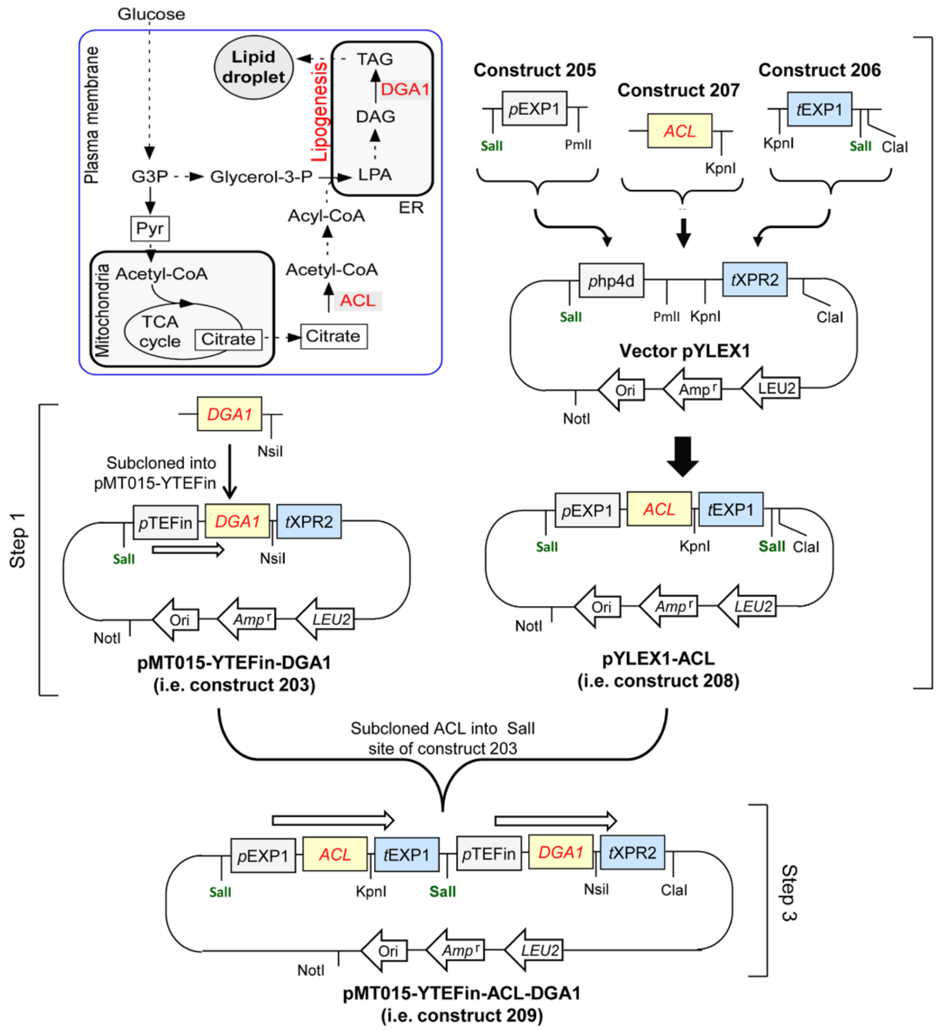

Supplementary Figure S1. A three-step process for constructing plasmids pYLEX1-ACL, pMT015-TEFin-DGA1 and pMT015-TEFin-DGA1-ACL. The roles of ACL and DGA1 in lipogenesis are shown in the left-top insert diagram and highlighted in red color. The generated plasmids were followed by transforming into Po1g URA3^{-} cells to obtain transformants of Po1g ACL, Po1g DGA1, and Po1g ACL-DGA1, respectively. See abbreviation section for the acronyms of genes and their components.

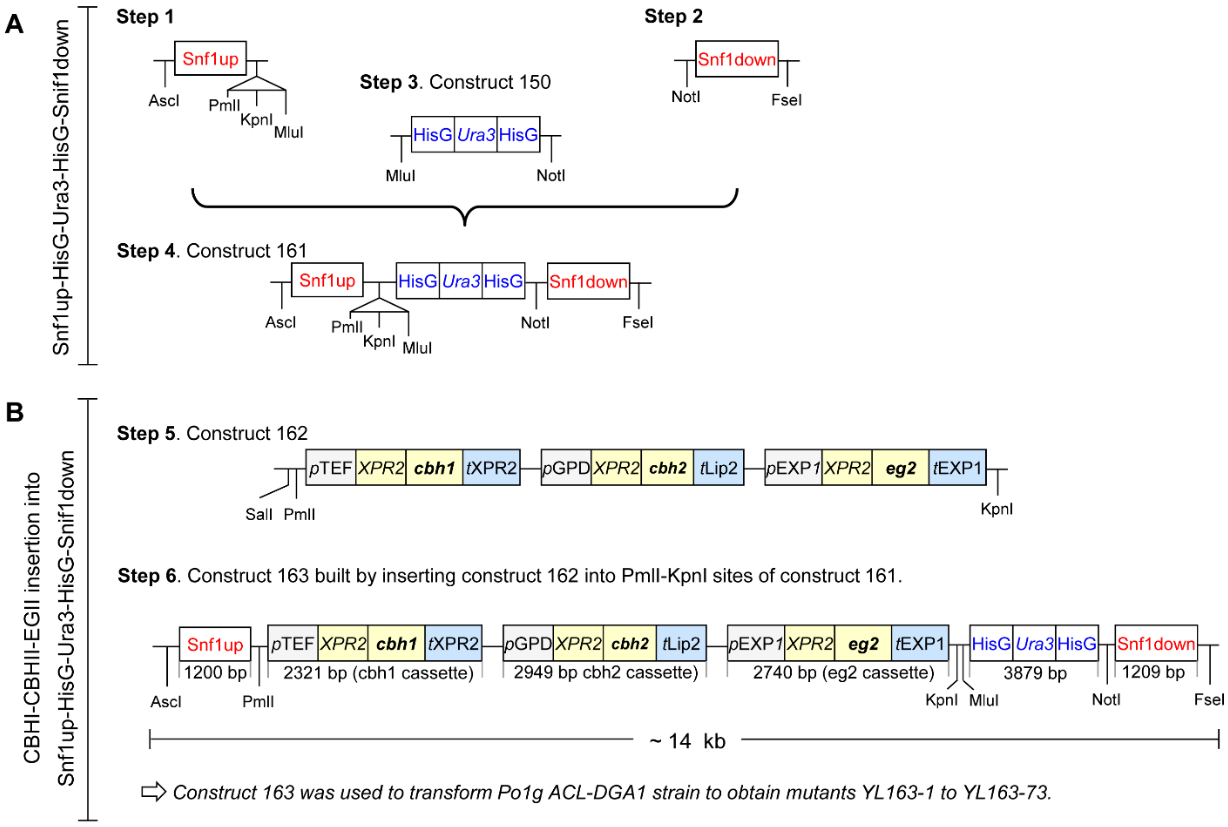

Supplementary Figure S2. Main steps in building the constructs for *Snf1* depletion, recyclable *URA3* marker and cellulase expression in *Y. lipolytica*. The details are described in the Materials and Methods section with construct sequences being listed in this additional file 1. The vector for each plasmid construct is also briefly described in Table 1. In steps 5 and 6, TEFin promoter and XPR2 terminator for Tr *cbh1* gene; GPD promoter and Lip2 terminator for Tr *cbh2*; EXP1 promoter and EXP1 terminator for Tr *eg2*, respectively. Other symbols and abbreviations: p, promoter; Snf1down, 1.2 kb upstream downstream nucleotide sequences of *Snf1* gene; Snf1up, 1.2 kb upstream nucleotide sequences of *Snf1* gene; t, terminator; XPR2, alkaline extracellular protease 2 pre-region (signal sequence).

Nucleotide sequences SEQ NO 1 to 11

SEQ ID NO 1

Promoter sequence of SalI-pEXP1-PmlI (1014 bp).

Note: SalI site (GTCGAC) at the 5' end; PmlI site (CACGTG) at the 3' end; start codon (ATG) for the downstream gene prior to the pmlI site. Cloning vector was pUC57.

GTCGACGAGTTTGGCGCCCGTTTTTCGAGCCCCACACGTTTCGGTGAGTATGAGCGGCGGC
AGATTCGAGCGTTTCCGGTTTCCGCGGCTGGACGAGAGCCCATGATGGGGGCTCCCACCACC
AGCAATCAGGGCCCTGATTACACACCCACCTGTAATGTCATGCTGTTCATCGTGGTTAATGC
TGCTGTGTGCTGTGTGTGTGTGTTGTTTGGCGCTCATTGTTGCGTTATGCAGCGTACACCACA
ATATTGGAAGCTTATTAGCCTTTCTATTTTTTCGTTTGCAAGGCTTAACAACATTGCTGTGGA
GAGGGATGGGGATATGGAGGCCGCTGGAGGGAGTCGGAGAGGCGTTTTGGAGCGGCTTGGC
CTGGCGCCCAGCTCGCGAAACGCACCTAGGACCCTTTGGCACGCCGAAATGTGCCACTTTTC
AGTCTAGTAACGCCTTACCTACGTCATTCCATGCATGCATGTTTGCGCCTTTTTTCCCTTGCCC
TTGATCGCCACACAGTACAGTGCACTGTACAGTGGAGGTTTTGGGGGGGTCTTAGATGGGAG
CTAAAAGCGGCCTAGCGGTACACTAGTGGGATTGTATGGAGTGGCATGGAGCCTAGGTGGA
GCCTGACAGGACGCACGACCGGCTAGCCCGTGACAGACGATGGGTGGCTCCTGTTGTCCACC
GCGTACAAATGTTTGGGCCAAAGTCTTGTCAGCCTTGCTTGCGAACCTAATTCCCAATTTTGT
CACTTCGCACCCCCATTGATCGAGCCCTAACCCCTGCCCATCAGGCAATCCAATTAAGCTCG
CATTGTCTGCCTTGTTTAGTTTGGCTCCTGCCCGTTTCGGCGTCCACTTGCACAAACACAAAC
AAGCATTATATATAAGGCTCGTCTCTCCCTCCCAACCACACTCACTTTTTTGCCCGTCTTCCC
TTGCTAACACAAAAGTCAAGAACACAAACAACCACCCCAACCCCCTTACACACAAGACATA
TCTACAGCAATGCACGTG,

SEQ NO 2

Terminator sequence of KpnI-tEXP1–SalI-ClaI (517 bp)

Note: KpnI site (GGTACC) at 5' end, and SalI and ClaI sites (GTCGACATCGAT) at the 3' end. Cloning vector was pUC57.

GGTACCAGCTTTGCGGCGAAACTCGATTCTCACCCTCGATAACTCGACTCACCCCCCTTAACT
TAAAATTCACTTACGACAAACAAACGTCTGATACCGACTACCCCCTCGACTTCTCGCAATCT
CGACTTTCAATCAGAGGACCTCAAACAACCACTTTTTTCTTACGATTCTAATTATTTACCCAT
TCATTAATTTCCCGTGCGCTCGTCCAGCAATGTCCGAGAGCATCCTGCCTGTGTCTCTGGGCC
CATCCATTTAATTTGGGTCCATCCTCCCGGGCAGATTCCACAGTCCAGTTGTCGCCGACTGGA
TGGTTAGTAGATCCGCCTTTTTTAGTTTGAACATGTTGCGAGTTGATACCTGAACATCAGAGT
TTTAGCTTCTTGTACTAATATACATTCCTTTTAGTGAGGTCCTGTACACTGTACACTGTATTAT
ATTGTAGCGTTGTATTTTTGATCCCTTGGTTTGTTCGTTATACGGTGTACAAGTATGTACGAT
CGTCGACATCGAT,

SEQ NO 3

Construct 207 for gene ACL (3312 bp)

Note: It is mouse ACL gene with codon optimization for expression in *Yarrowia*. It had blunt end at 5' end, and stop codon and KpnI at 3' end; the start codon is at the 3' end of above "SalI-pEXP1-PmlI" sequence prior to the pmlI site Cloning vector PUC57

TCCGCTAAGGCTATCTCCGAGCAGACTGGAAAGGAGCTGCTCTACAAGTACATTTGCACTAC
CTCCGCCATTCAGAACCGATTCAAGTACGCCCGAGTGACCCCCGACACTGATTGGGCTCACC
TGCTCCAGGACCATCCCTGGCTGCTCTCCCAGTCGCTGGTGGTCAAGCCTGATCAGCTCATC
AAGCGACGAGGCAAGCTGGGACTCGTCGGTGTTAACCTGTCGCTCGACGGCGTCAAGTCTTG
GCTGAAGCCTCGACTCGGACACGAGGCTACTGTGGGCAAGGCTAAGGGCTTCCTGAAGAAC
TTTCTCATCGAGCCCTTCGTTCCTCATTCCCAGGCCGAGGAGTTTTACGTGTGTATCTACGCT
ACCCGAGAGGGCGACTACGTCCTGTTCCACCATGAGGGCGGAGTGGATGTCGGCGACGTTG
ATGCTAAGGCCCAGAAGCTGCTCGTTGGAGTGGACGAGAAGCTGAACACCGAGGATATCAA
GCGACACCTGCTCGTTCATGCCCCCGAGGACAAGAAGGAGGTGCTGGCTTCGTTCATTTCTG
GCCTCTTCAACTTTTACGAGGATCTCTACTTTACTTACCTGGAGATCAACCCTCTCGTTGTGA
CCAAGGACGGAGTCTACATTCTGGATCTCGCCGCTAAGGTTGACGCTACCGCCGATTACATC
TGTAAGGTGAAGTGGGGTGACATTGAGTTCCCTCCTCCTTTTGGCCGAGAGGCTTACCCTGA
GGAGGCTTACATCGCTGACCTGGATGCCAAGTCCGGTGCTTCGCTGAAGCTCACTCTGCTCA
ACCCTAAGGGCCGAATCTGGACCATGGTCGCTGGTGGTGGAGCTTCGGTCGTTTACTCTGAC
ACCATTTGCGATCTGGGTGGCGTGAACGAGCTCGCCAACTACGGAGAGTACTCCGGTGCTCC
CTCGGAGCAGCAGACCTACGACTACGCCAAGACTATCCTGTCCCTCATGACCCGAGAGAAG
CACCCTGAGGGCAAGATCCTGATCATTGGAGGTTCGATTGCTAACTTCACCAACGTGGCCGC
TACTTTTAAGGGTATCGTCCGAGCCATTCGAGACTACCAGGGCCCCCTGAAGGAGCATGAGG
TGACCATTTTCGTCCGACGAGGCGGACCTAACTACCAGGAGGGACTCCGAGTTATGGGCGA
AGTGGGAAAGACCACTGGCATCCCCATTCACGTTTTCGGAACCGAGACTCATATGACCGCCA
TCGTGGGCATGGCTCTGGGACACCGACCCATTCCTAACCAGCCTCCCACCGCCGCTCATACT
GCCAACTTCCTGCTCAACGCTTCTGGCTCCACCTCGACTCCTGCTCCTTCTCGAACCGCTTCT
TTTTCCGAGTCTCGAGCTGACGAGGTCGCTCCTGCCAAGAAGGCTAAGCCTGCTATGCCTCA
GGATTCTGTGCCCTCCCCTCGATCGCTGCAGGGAAAGTCGGCCACTCTCTTCTCTCGACACAC
CAAGGCTATCGTTTGGGGCATGCAGACTCGAGCCGTGCAGGGTATGCTGGACTTTGATTACG
TCTGCTCCCGAGACGAGCCTTCGGTCGCTGCTATGGTTTACCCTTTCACCGGAGATCACAAG
CAGAAGTTTTACTGGGGTCATAAGGAGATCCTGATTCCCGTGTTCAAGAACATGGCTGACGC
CATGAAGAAGCACCCTGAGGTCGATGTTCTGATTAACTTTGCCTCTCTCCGATCCGCTTACGA
CTCCACCATGGAGACTATGAACTACGCCCAGATCCGAACTATTGCCATCATTGCTGAGGGCA
TCCCCGAGGCTCTGACCCGAAAGCTCATTAAGAAGGCCGACCAGAAGGGTGTCACTATCATT
GGCCCCGCCACCGTTGGTGGCATCAAGCCTGGATGTTTCAAGATTGGTAACACCGGCGGTAT
GCTGGACAACATCCTCGCTTCCAAGCTGTACCGACCCGGCTCGGTTGCCTACGTGTCTCGAT
CCGGCGGAATGTCTAACGAGCTGAACAACATCATTTCCCGAACCACTGACGGTGTGTACGAG
GGCGTCGCCATTGGTGGCGACCGATACCCCGGATCTACTTTCATGGACCACGTGCTGCGATA
CCAGGATACCCCTGGCGTCAAGATGATCGTGGTCCTCGGAGAGATTGGAGGTACTGAGGAG
TACAAGATCTGCCGAGGAATTAAGGAGGGCCGACTGACCAAGCCCGTTGTGTGTTGGTGCAT
CGGAACCTGTGCTACTATGTTCTCTTCCGAGGTCCAGTTTGGTCACGCTGGTGCTTGCGCTAA

CCAGGCTTCCGAGACCGCTGTGGCTAAGAACCAGGCCCTGAAGGAGGCTGGAGTGTTCGTC
CCTCGATCGTTTGACGAGCTGGGAGAGATCATTCAGTCTGTTTACGAGGATCTCGTGGCCAA
GGGCGCTATTGTCCCTGCTCAGGAGGTTCCTCCTCCTACCGTGCCTATGGACTACTCTTGGGC
TCGAGAGCTGGGACTCATCCGAAAGCCCGCCTCTTTCATGACTTCCATTTGTGACGAGCGGG
GTCAGGAGCTGATCTACGCCGGCATGCCTATTACCGAGGTCTTTAAGGAGGAGATGGGAATC
GGCGGAGTTCTGGGTCTGCTCTGGTTCCAGCGACGACTCCCCAAGTACTCTTGTCAGTTTATT
GAGATGTGCCTGATGGTGACTGCTGACCACGGTCCTGCCGTCTCCGGAGCTCATAACACCAT
CATTTGCGCCCGAGCTGGAAAGGACCTCGTGTCGTCTCTGACCTCCGGTCTGCTCACTATCG
GCGACCGATTCGGTGGCGCTCTGGATGCCGCTGCCAAGATGTTCTCTAAGGCCTTTGACTCC
GGAATCATTCCCATGGAGTTCGTCAACAAGATGAAGAAGGAGGGCAAGCTGATCATGGGAA
TTGGTCACCGAGTGAAGTCCATCAACAACCCTGACATGCGAGTCCAGATTCTGAAGGATTTC
GTTAAGCAGCATTTTCCCGCCACCCCTCTGCTCGACTACGCTCTCGAGGTCGAGAAGATCAC
CACTTCTAAGAAGCCCAACCTGATCCTCAACGTGGACGGATTCATTGGTGTCGCCTTTGTTG
ATATGCTGCGAAACTGTGGCTCGTTCACCCGAGAGGAGGCCGACGAGTACGTGGATATCGG
CGCTCTGAACGGAATTTTCGTCCTCGGACGATCTATGGGCTTTATTGGACACTACCTGGACC
AGAAGCGACTCAAGCAGGGTCTCTACCGACACCCTTGGGATGATATTTCTTACGTCCTGCCT
GAGCACATGTCTATGTAATAGGGTACC,

SEQ NO 4

Full sequence of vector pMT015-YTEFin was described in literature (Tai and Stephanopoulos, 2013).

SEQ NO 5

DGA1 gene synthesized (1560 bp), with a **5' b****lunt end and a NsiI site on the 3' end**

TAACCGCAGACTATCGACTCACAATACTACAAGTCGCGAGACAAAAACGACACGGCACCCA
AAATCGCGGGAATCCGATATGCCCCGCTATCGACACCATTACTCAACCGATGTGAGACCTTC
TCTCTGGTCTGGCACATTTTCAGCATTCCCACTTTCCTCACAATTTTCATGCTATGCTGCGCA
ATTCCACTGCTCTGGCCATTTGTGATTGCGTATGTAGTGTACGCTGTTAAAGACGACTCCCCG
TCCAACGGAGGAGTGGTCAAGCGATACTCGCCTATTTCAAGAAACTTCTTCATCTGGAAGCT
CTTTGGCCGCTACTTCCCCATAACTCTGCACAAGACGGTGGATCTGGAGCCCACGCACACAT
ACTACCCTCTGGACGTCCAGGAGTATCACCTGATTGCTGAGAGATACTGGCCGCAGAACAAG
TACCTCCGAGCAATCATCTCCACCATCGAGTACTTTCTGCCCGCCTTCATGAAACGGTCTCTT
TCTATCAACGAGCAGGAGCAGCCTGCCGAGCGAGATCCTCTCCTGTCTCCCGTTTCTCCCAG
CTCTCCGGGTTCTCAACCTGACAAGTGGATTAACCACGACAGCAGATATAGCCGTGGAGAAT
CATCTGGCTCCAACGGCCACGCCTCGGGCTCCGAACTTAACGGCAACGGCAACAATGGCAC
CACTAACCGACGACCTTTGTCGTCCGCCTCTGCTGGCTCCACTGCATCTGATTCCACGCTTCT
TAACGGGTCCCTCAACTCCTACGCCAACCAGATCATTGGCGAAAACGACCCACAGCTGTCGC
CCACAAAACTCAAGCCCACTGGCAGAAAATACATCTTCGGCTACCACCCCCACGGCATTATC
GGCATGGGAGCCTTTGGTGGAATTGCCACCGAGGGAGCTGGATGGTCCAAGCTCTTTCCGGG
CATCCCTGTTTCTCTTATGACTCTCACCAACAACTTCCGAGTGCCTCTCTACAGAGAGTACCT
CATGAGTCTGGGAGTCGCTTCTGTCTCCAAGAAGTCCTGCAAGGCCCTCCTCAAGCGAAACC
AGTCTATCTGCATTGTCGTTGGTGGAGCACAGGAAAGTCTTCTGGCCAGACCCGGTGTCATG
GACCTGGTGCTACTCAAGCGAAAGGGTTTTGTTCGACTTGGTATGGAGGTCGGAAATGTCGC

CCTTGTTCCCATCATGGCCTTTGGTGAGAACGACCTCTATGACCAGGTTAGCAACGACAAGT
CGTCCAAGCTGTACCGATTCCAGCAGTTTGTCAAGAACTTCCTTGGATTCACCCTTCCTTTGA
TGCATGCCCGAGGCGTCTTCAACTACGATGTCGGTCTTGTCCCCTACAGGCGACCCGTCAAC
ATTGTGGTTGGTTCCCCCATTGACTTGCCTTATCTCCCACACCCCACCGACGAAGAAGTGTCC
GAATACCACGACCGATACATCGCCGAGCTGCAGCGAATCTACAACGAGCACAAGGATGAAT
ATTTCATCGATTGGACCGAGGAGGGCAAAGGAGCCCCAGAGTTCCGAATGATTGAGTAATA
GATGCAT

SEQ NO 6

SNFup sequence: (length: 1238 bp). Cloning vector name: pUC57. It contains:

(1) AscI (GGCGCGCC)

(2) The *Yarrowia Snf1* upstream (base 234933 to 236132 of *Yarrowia lipolytica* CLIB122 chromosome D complete sequence; 1200 bp)

(3) PmlI (CACGTG), KpnI (GGTACC), and MluI (ACGCGT)

GGCGCGCCTGATTACACCCTGGAACTGTTGGGTGGAGACAAGCCAGATATTCGGGAGATCC
AGCTGAATCTGAACGGGTTTCTGGAGGAGAACACAGCCAAGTTCTGCAAGGAGCTGTGGGA
GCTGCTGGTGGCTGCCCAGAAAGACAAGGACGGCATTCCGCCGCAGTTGATTGCCATCAAG
AAGGAGCAGATGGAGCAGGAGCGGGTGAAGCGGGAAATCAAGATTGAGAGTTGGAGTGGT
GGGGGCGGTGGTGGAAGAGACAGAGGTGATAGAAGGGAAAGAAACGACAGAAGAGACGC
AAAAGGAAGAAACAGAAGAGATGGAGATAGGAGGAATGGCGATCGAAGAGACAGAGACA
GAGATAGAGATAGAGACGACAGGTCAAGTCGTTCAAGTCGGTACAACCGATCAAGATCAAG
ATCTCCGACTGTCAAAAAGGAAACAGATGAATATGGCCGGGACCGGAAGGACTAACTGCAT
TATATATAAATATAGATTCTTGATTAGTGATCAAAGCATGAGATACTGTCAAGGCAAGCAAG
CCTTACAATATCTGTTGGCTTAGTTAAATCTTTTTTGAGATGTAATAATTGGGGAACCTCTGT
TAGATGAACAGTTGCGGCTTACTCATCTTAGACATGGTCCAGTTTAGGCGTAATTAATTATC
AGTTTTAATGCGCCTCTACGTAATAAGCGTTGCCACAAGTTACAGTAGAGATGATACCATTG
CTACAGGACCGTATAGCGTATATAATGACGAGGCTAATTGATGATGATCGACAGCGAAGAC
ATATCTACGTCAATCTGACGCTTCATTTTGTGCTTCTTGTTCGGATTTGTGTTTCTCCCAGTGC
ACCCAGTTTTTGCTGACCATTTTTTGCTTACCAGTTTTTGCTCGCGCGTTTTCTCGATTGAAC
TCTGCTGCGAGGGTGGTGTAAGACACACTCCCTGTAGAACATGCATTTCGAGGTGTGACATT
TCCATCTCTGTTAAATCAAACGAGTGACACAAACGGAATAAATCTACATATACGTCGAACCC
TCTTCTTCTCTTCTCTCTCTTCCTTAATAGACACCCTCGCTATCGACTCTGCTCCTCCTTATCA
CACCATCAAAGTTTAGGGTGTGTCCCAACACCACCTCCACACCACACTCTATTATCACCACC
TCCACGACTACATACCACTCCTTCCACCACCTCACAACACGTGAAGCTTCTAGAGGTACCAC
GCGTA,

SEQ NO 7

Snf1down Sequence (Length: 1217 bp); Vector name: pUC57.

GCGGCCGCGCACTTGTAGAGCACACTAGGGATTTAGAGGGGATTATGGCACGTACAATATA
GATAATTAAGCAGTAGCTGAGTCAGTTGAATGATCAGAGGTGTAACATGAGTGTGGATGGA
TTGTGTAGAGTCGTTGTTTAAAATAATGAGTTAAGAATAATTATACGACTACAGGATACGAT
GTACTTGTATTGTATCGATACAGTACATACAGTACATACGTGTAACATACTCCTAAACTGTTG
CATCACCTACAACTCCAACTAGTCGGTCATAATTCATTAATACGTTTCCCTGGTGTAGTCTAG
GCCAAACACTCCTTGGTGAACTTCTTGAGAAAGATTCGCTCTCTAGACTCCTTGAATTCTCTA
GGCTGGATTTCGGCCCAAAAAGAACCCACAAACGGCGCAATACTCTTCTTGTAGCCGGAATA
GTTGTCTCGAATGACAATATCCGGGTTGGCAGACCGCTGCTGATGCTCCTTGACGATGATAC
TCTCAAAGTGGTCCACGGTAGACATGACAATAAGAGCCGACGAGGTTTTGCTCTTGTAGTTG
GCGATGAGCGTTTTAAGTGCCCCAGAGTTCTCCACCACCTTGTGGATCAGCTGATGCTTTTGT
TCCTCGCTGAGTCCTTCCGAGGCCAGGGGGATAGCCAAGCAGGTGAGAATAGCGCCTGTGTC
TCGTTTAGAAGAGGTGCTGTTTGTATCATCCGCATGCTTGTTGATGAACTGAACTAAATTCAG
TAGCGTGCTCCACAGCTCATTCCAGTGGTAATTTGAGGGAGTGTCCCTGTAAACCATGAGTG
TTGCTTCAGTGACGACCAGAGCGAGATCATAAATGTCTGGGAGAGGTTTCTTCATATTGTAT
CTGAGACAGCAAAGAAGTGCGTCCAAAAGGCCGGTTCCAAAGGTGTAGGCCTCTGTTTCTG
GAAGGGGGGGTTTCCGTTGCTTGGCGTTGATTTTAATAGATGCTTTTGTGTTGAACGACTGGT
GCAATTCGGGCAGAAGAAGCTGCAGAATGATTAGAGCCACCTTACTGTACATTTCAGCTCGT
GGGTTTTTGTTTTGGTTGGAAAAGAGGTACGAACAGAGAGAGAGAAAGGCTGCCAGGGCAG
GCTCGGTGTCATAGTTCTTGGTACGATGTCCTTTATCGGTAATGAGTGTCTTTGCAAACAACT
TGTTCTTCTGCACGAATTCCAAAGTGGGGGCCGGCC,

SEQ NO 8

Construct pNREL150, *i.e.,* HisG-*URA3*-HisG (length: 3885 bp). Cloning vector: pUC57. Cloning site: BamHI-HindIII.

BamHI site GGATCC (5' end, position 1 to 6); MluI site ACGCGT + A (7 to 13); HisG of *S. typhimurium* (position 14 to 1169); *URA3* gene cassette (1170 to 2715); HisG of *S. typhimurium* (2716 to 3871); HindIII site AAGCTT (3' end, 3880 to 3885); NotI site GCGGCCGC (3872 to 3879).

GGATCCACGCGTAGATCTTCCAGTGGTGCATGAACGCATGAGAAAGCCCCCGGAAGATCAT
CTTCCGGGGGCTTTTTTTTTGGCGCGCGATACAGACCGGTTCAGACAGGATAAAGAGGAACG
CAGAATGTTAGACAACACCCGCTTACGCATAGCTATTCAGAAATCAGGCCGTTTAAGCGATG
ATTCACGAGAATTGCTGGCCCGCTGCGGCATAAAAATTAATTTACACACTCAGCGCCTGATT
GCGATGGCGGAAAACATGCCGATTGATATCCTGCGCGTGCGTGATGATGACATTCCGGGTCT
GGTAATGGATGGCGTGGTCGATCTCGGTATTATCGGCGAAAACGTGCTGGAAGAAGAGCTA
CTCAACCGCCGCGCACAGGGCGAAGATCCACGCTATTTAACCCTGCGCCGTCTTGACTTCGG
CGGCTGCCGTTTATCGCTGGCAACACCGGTTGACGAAGCCTGGGACGGCCCGGCCGCGCTGG
ACGGTAAACGTATCGCTACCTCATATCCGCACCTCCTCAAACGCTACCTCGACCAGAAAGGC
GTCTCTTTTAAATCGTGTCTGTTAAATGGTTCTGTCGAAGTCGCGCCGCGCGCGGGGCTGGCC
GACGCTATCTGCGATTTGGTCTCTACCGGCGCGACGCTTGAAGCTAACGGCCTGCGTGAAGT
CGAAGTTATCTACCGCTCTAAAGCCTGTCTGATTCAGCGCGACGGTGAGATGGCACAGAGCA

AGCAAGAGCTGATCGATAAATTGCTGACCCGTATTCAGGGCGTGATTCAGGCGCGCGAATC
GAAATACATCATGATGCACGCGCCAAGTGAACGCCTGGAAGAGGTTATCGCCCTGCTGCCA
GGCGCCGAAAGGCCGACAATTCTGCCGCTGGCAGGCGAGCAACAGCGCGTGGCGATGCACA
TGGTCAGCAGCGAAACGTTGTTCTGGGAAACCATGGAGAAACTGAAAGCGCTTGGCGCCAG
CTCGATTCTGGTACTGCCGATCGAGAAGATGATGGAGTGATCTGACGCCTGATGGCGCTGCG
CTTATCAGGCCTACGTAATGCGTTGATATTTTGGGTTCTGTAGGCCGGATAAGGCGGAACCC
TGTGATGGAGTAAAGACCATGAGCTTCAATACCCTGATTGACTGGAACAGCGGATCTGGTCG
ACGAGTATCTGTCTGACTCGTCATTGCCGCCTTTGGAGTACGACTCCAACTATGAGTGTGCTT
GGATCACTTTGACGATACATTCTTCGTTGGAGGCTGTGGGTCTGACAGCTGCGTTTTCGGCGC
GGTTGGCCGACAACAATATCAGCTGCAACGTCATTGCTGGCTTTCATCATGATCACATTTTTG
TCGGCAAAGGCGACGCCCAGAGAGCCATTGACGTTCTTTCTAATTTGGACCGATAGCCGTAT
AGTCCAGTCTATCTATAAGTTCAACTAACTCGTAACTATTACCATAACATATACTTCACTGCC
CCAGATAAGGTTCCGATAAAAAGTTCTGCAGACTAAATTTATTTCAGTCTCCTCTTCACCACC
AAAATGCCCTCCTACGAAGCTCGAGCTAACGTCCACAAGTCCGCCTTTGCCGCTCGAGTGCT
CAAGCTCGTGGCAGCCAAGAAAACCAACCTGTGTGCTTCTCTGGATGTTACCACCACCAAGG
AGCTCATTGAGCTTGCCGATAAGGTCGGACCTTATGTGTGCATGATCAAGACCCATATCGAC
ATCATTGACGACTTCACCTACGCCGGCACTGTGCTCCCCCTCAAGGAACTTGCTCTTAAGCA
CGGTTTCTTCCTGTTCGAGGACAGAAAGTTCGCAGATATTGGCAACACTGTCAAGCACCAGT
ACAAGAACGGTGTCTACCGAATCGCCGAGTGGTCCGATATCACCAACGCCCACGGTGTACCC
GGAACCGGAATCATTGCTGGCCTGCGAGCTGGTGCCGAGGAAACTGTCTCTGAACAGAAGA
AGGAGGACGTCTCTGACTACGAGAACTCCCAGTACAAGGAGTTCCTGGTCCCCTCTCCCAAC
GAGAAGCTGGCCAGAGGTCTGCTCATGCTGGCCGAGCTGTCTTGCAAGGGCTCTCTGGCCAC
TGGCGAGTACTCCAAGCAGACCATTGAGCTTGCCCGATCCGACCCCGAGTTTGTGGTTGGCT
TCATTGCCCAGAACCGACCTAAGGGCGACTCTGAGGACTGGCTTATTCTGACCCCCGGGGTG
GGTCTTGACGACAAGGGAGACGCTCTCGGACAGCAGTACCGAACTGTTGAGGATGTCATGT
CTACCGGAACGGATATCATAATTGTCGGCCGAGGTCTGTACGGCCAGAACCGAGATCCTATT
GAGGAGGCCAAGCGATACCAGAAGGCTGGCTGGGAGGCTTACCAGAAGATTAACTGTTAGA
GGTTAGACTATGGATATGTCATTTAACTGTGTATATAGAGAGCGTGCAAGTATGGAGCGCTT
GTTCAGCTTGTATGATGGTCAGACGACCTGTCTGATCGAGTATGTATGATACTGCACAACCT
GTGTATCCGCATGATCTGTCCAATGGGGCATGTTGTTGTGTTTCTCGATACGGAGATGCTGG
GTACAAGTAGCTAATACGATTGAACTACTTATACTTATATGAGGCTTGAAGAAAGCTGACTT
GTGTATGACTTATTCTCAACTACATCCCCAGTCACAATACCACCACTGCACGGATCTTCCAGT
GGTGCATGAACGCATGAGAAAGCCCCCGGAAGATCATCTTCCGGGGGCTTTTTTTTTGGCGC
GCGATACAGACCGGTTCAGACAGGATAAAGAGGAACGCAGAATGTTAGACAACACCCGCTT
ACGCATAGCTATTCAGAAATCAGGCCGTTTAAGCGATGATTCACGAGAATTGCTGGCCCGCT
GCGGCATAAAAATTAATTTACACACTCAGCGCCTGATTGCGATGGCGGAAAACATGCCGATT
GATATCCTGCGCGTGCGTGATGATGACATTCCGGGTCTGGTAATGGATGGCGTGGTCGATCT
CGGTATTATCGGCGAAAACGTGCTGGAAGAAGAGCTACTCAACCGCCGCGCACAGGGCGAA
GATCCACGCTATTTAACCCTGCGCCGTCTTGACTTCGGCGGCTGCCGTTTATCGCTGGCAACA
CCGGTTGACGAAGCCTGGGACGGCCCGGCCGCGCTGGACGGTAAACGTATCGCTACCTCAT
ATCCGCACCTCCTCAAACGCTACCTCGACCAGAAAGGCGTCTCTTTTAAATCGTGTCTGTTAA
ATGGTTCTGTCGAAGTCGCGCCGCGCGCGGGGCTGGCCGACGCTATCTGCGATTTGGTCTCT
ACCGGCGCGACGCTTGAAGCTAACGGCCTGCGTGAAGTCGAAGTTATCTACCGCTCTAAAGC
CTGTCTGATTCAGCGCGACGGTGAGATGGCACAGAGCAAGCAAGAGCTGATCGATAAATTG

CTGACCCGTATTCAGGGCGTGATTCAGGCGCGCGAATCGAAATACATCATGATGCACGCGCC
AAGTGAACGCCTGGAAGAGGTTATCGCCCTGCTGCCAGGCGCCGAAAGGCCGACAATTCTG
CCGCTGGCAGGCGAGCAACAGCGCGTGGCGATGCACATGGTCAGCAGCGAAACGTTGTTCT
GGGAAACCATGGAGAAACTGAAAGCGCTTGGCGCCAGCTCGATTCTGGTACTGCCGATCGA
GAAGATGATGGAGTGATCTGACGCCTGATGGCGCTGCGCTTATCAGGCCTACGTAATGCGTT
GATATTTTGGGTTCTGTAGGCCGGATAAGGCGGAACCCTGTGATGGAGTAAAGACCATGAG
CTTCAATACCCTGATTGACTGGAACAGCGGATCTGGCGGCCGCAAGCTT,

SEQ NO 9

Construct 161 (length: 6313 bp) for Snf1up-HisG-*URA3*-HisG-Snf1down. Cloning vector: pUC57-Simple.

AscI site (GG^CGCG_CC) at the 5’ end; FseI site (GG_CCGG^CC) at the 3’ end.

GGCGCGCCTGATTACACCCTGGAACTGTTGGGTGGAGACAAGCCAGATATTCGGGAGATCC
AGCTGAATCTGAACGGGTTTCTGGAGGAGAACACAGCCAAGTTCTGCAAGGAGCTGTGGGA
GCTGCTGGTGGCTGCCCAGAAAGACAAGGACGGCATTCCGCCGCAGTTGATTGCCATCAAG
AAGGAGCAGATGGAGCAGGAGCGGGTGAAGCGGGAAATCAAGATTGAGAGTTGGAGTGGT
GGGGGCGGTGGTGGAAGAGACAGAGGTGATAGAAGGGAAAGAAACGACAGAAGAGACGC
AAAAGGAAGAAACAGAAGAGATGGAGATAGGAGGAATGGCGATCGAAGAGACAGAGACA
GAGATAGAGATAGAGACGACAGGTCAAGTCGTTCAAGTCGGTACAACCGATCAAGATCAAG
ATCTCCGACTGTCAAAAAGGAAACAGATGAATATGGCCGGGACCGGAAGGACTAACTGCAT
TATATATAAATATAGATTCTTGATTAGTGATCAAAGCATGAGATACTGTCAAGGCAAGCAAG
CCTTACAATATCTGTTGGCTTAGTTAAATCTTTTTTGAGATGTAATAATTGGGGAACCTCTGT
TAGATGAACAGTTGCGGCTTACTCATCTTAGACATGGTCCAGTTTAGGCGTAATTAATTATC
AGTTTTAATGCGCCTCTACGTAATAAGCGTTGCCACAAGTTACAGTAGAGATGATACCATTG
CTACAGGACCGTATAGCGTATATAATGACGAGGCTAATTGATGATGATCGACAGCGAAGAC
ATATCTACGTCAATCTGACGCTTCATTTTGTGCTTCTTGTTCGGATTTGTGTTTCTCCCAGTGC
ACCCAGTTTTTTGCTGACCATTTTTTGCTTACCAGTTTTTGCTCGCGCGTTTTCTCGATTGAAC
TCTGCTGCGAGGGTGGTGTAAGACACACTCCCTGTAGAACATGCATTTCGAGGTGTGACATT
TCCATCTCTGTTAAATCAAACGAGTGACACAAACGGAATAAATCTACATATACGTCGAACCC
TCTTCTTCTCTTCTCTCTCTTCCTTAATAGACACCCTCGCTATCGACTCTGCTCCTCCTTATCA
CACCATCAAAGTTTAGGGTGTGTCCCAACACCACCTCCACACCACACTCTATTATCACCACC
TCCACGACTACATACCACTCCTTCCACCACCTCACAACACGTGAAGCTTCTAGAGGTACCAC
GCGTAGATCTTCCAGTGGTGCATGAACGCATGAGAAAGCCCCCGGAAGATCATCTTCCGGG
GGCTTTTTTTTTGGCGCGCGATACAGACCGGTTCAGACAGGATAAAGAGGAACGCAGAATGT
TAGACAACACCCGCTTACGCATAGCTATTCAGAAATCAGGCCGTTTAAGCGATGATTCACGA
GAATTGCTGGCCCGCTGCGGCATAAAAATTAATTTACACACTCAGCGCCTGATTGCGATGGC
GGAAAACATGCCGATTGATATCCTGCGCGTGCGTGATGATGACATTCCGGGTCTGGTAATGG
ATGGCGTGGTCGATCTCGGTATTATCGGCGAAAACGTGCTGGAAGAAGAGCTACTCAACCG
CCGCGCACAGGGCGAAGATCCACGCTATTTAACCCTGCGCCGTCTTGACTTCGGCGGCTGCC
GTTTATCGCTGGCAACACCGGTTGACGAAGCCTGGGACGGCCCGGCCGCGCTGGACGGTAA
ACGTATCGCTACCTCATATCCGCACCTCCTCAAACGCTACCTCGACCAGAAAGGCGTCTCTTT

TAAATCGTGTCTGTTAAATGGTTCTGTCGAAGTCGCGCCGCGCGCGGGGCTGGCCGACGCTA
TCTGCGATTTGGTCTCTACCGGCGCGACGCTTGAAGCTAACGGCCTGCGTGAAGTCGAAGTT
ATCTACCGCTCTAAAGCCTGTCTGATTCAGCGCGACGGTGAGATGGCACAGAGCAAGCAAG
AGCTGATCGATAAATTGCTGACCCGTATTCAGGGCGTGATTCAGGCGCGCGAATCGAAATAC
ATCATGATGCACGCGCCAAGTGAACGCCTGGAAGAGGTTATCGCCCTGCTGCCAGGCGCCG
AAAGGCCGACAATTCTGCCGCTGGCAGGCGAGCAACAGCGCGTGGCGATGCACATGGTCAG
CAGCGAAACGTTGTTCTGGGAAACCATGGAGAAACTGAAAGCGCTTGGCGCCAGCTCGATT
CTGGTACTGCCGATCGAGAAGATGATGGAGTGATCTGACGCCTGATGGCGCTGCGCTTATCA
GGCCTACGTAATGCGTTGATATTTTGGGTTCTGTAGGCCGGATAAGGCGGAACCCTGTGATG
GAGTAAAGACCATGAGCTTCAATACCCTGATTGACTGGAACAGCGGATCTGGTCGACGAGT
ATCTGTCTGACTCGTCATTGCCGCCTTTGGAGTACGACTCCAACTATGAGTGTGCTTGGATCA
CTTTGACGATACATTCTTCGTTGGAGGCTGTGGGTCTGACAGCTGCGTTTTCGGCGCGGTTGG
CCGACAACAATATCAGCTGCAACGTCATTGCTGGCTTTCATCATGATCACATTTTTGTCGGCA
AAGGCGACGCCCAGAGAGCCATTGACGTTCTTTCTAATTTGGACCGATAGCCGTATAGTCCA
GTCTATCTATAAGTTCAACTAACTCGTAACTATTACCATAACATATACTTCACTGCCCCAGAT
AAGGTTCCGATAAAAAGTTCTGCAGACTAAATTTATTTCAGTCTCCTCTTCACCACCAAAAT
GCCCTCCTACGAAGCTCGAGCTAACGTCCACAAGTCCGCCTTTGCCGCTCGAGTGCTCAAGC
TCGTGGCAGCCAAGAAAACCAACCTGTGTGCTTCTCTGGATGTTACCACCACCAAGGAGCTC
ATTGAGCTTGCCGATAAGGTCGGACCTTATGTGTGCATGATCAAGACCCATATCGACATCAT
TGACGACTTCACCTACGCCGGCACTGTGCTCCCCCTCAAGGAACTTGCTCTTAAGCACGGTTT
CTTCCTGTTCGAGGACAGAAAGTTCGCAGATATTGGCAACACTGTCAAGCACCAGTACAAGA
ACGGTGTCTACCGAATCGCCGAGTGGTCCGATATCACCAACGCCCACGGTGTACCCGGAACC
GGAATCATTGCTGGCCTGCGAGCTGGTGCCGAGGAAACTGTCTCTGAACAGAAGAAGGAGG
ACGTCTCTGACTACGAGAACTCCCAGTACAAGGAGTTCCTGGTCCCCTCTCCCAACGAGAAG
CTGGCCAGAGGTCTGCTCATGCTGGCCGAGCTGTCTTGCAAGGGCTCTCTGGCCACTGGCGA
GTACTCCAAGCAGACCATTGAGCTTGCCCGATCCGACCCCGAGTTTGTGGTTGGCTTCATTG
CCCAGAACCGACCTAAGGGCGACTCTGAGGACTGGCTTATTCTGACCCCCGGGGTGGGTCTT
GACGACAAGGGAGACGCTCTCGGACAGCAGTACCGAACTGTTGAGGATGTCATGTCTACCG
GAACGGATATCATAATTGTCGGCCGAGGTCTGTACGGCCAGAACCGAGATCCTATTGAGGA
GGCCAAGCGATACCAGAAGGCTGGCTGGGAGGCTTACCAGAAGATTAACTGTTAGAGGTTA
GACTATGGATATGTCATTTAACTGTGTATATAGAGAGCGTGCAAGTATGGAGCGCTTGTTCA
GCTTGTATGATGGTCAGACGACCTGTCTGATCGAGTATGTATGATACTGCACAACCTGTGTA
TCCGCATGATCTGTCCAATGGGGCATGTTGTTGTGTTTCTCGATACGGAGATGCTGGGTACA
AGTAGCTAATACGATTGAACTACTTATACTTATATGAGGCTTGAAGAAAGCTGACTTGTGTA
TGACTTATTCTCAACTACATCCCCAGTCACAATACCACCACTGCACGGATCTTCCAGTGGTGC
ATGAACGCATGAGAAAGCCCCCGGAAGATCATCTTCCGGGGGCTTTTTTTTTGGCGCGCGAT
ACAGACCGGTTCAGACAGGATAAAGAGGAACGCAGAATGTTAGACAACACCCGCTTACGCA
TAGCTATTCAGAAATCAGGCCGTTTAAGCGATGATTCACGAGAATTGCTGGCCCGCTGCGGC
ATAAAAATTAATTTACACACTCAGCGCCTGATTGCGATGGCGGAAAACATGCCGATTGATAT
CCTGCGCGTGCGTGATGATGACATTCCGGGTCTGGTAATGGATGGCGTGGTCGATCTCGGTA
TTATCGGCGAAAACGTGCTGGAAGAAGAGCTACTCAACCGCCGCGCACAGGGCGAAGATCC
ACGCTATTTAACCCTGCGCCGTCTTGACTTCGGCGGCTGCCGTTTATCGCTGGCAACACCGGT
TGACGAAGCCTGGGACGGCCCGGCCGCGCTGGACGGTAAACGTATCGCTACCTCATATCCGC
ACCTCCTCAAACGCTACCTCGACCAGAAAGGCGTCTCTTTTAAATCGTGTCTGTTAAATGGTT

CTGTCGAAGTCGCGCCGCGCGCGGGGCTGGCCGACGCTATCTGCGATTTGGTCTCTACCGGC
GCGACGCTTGAAGCTAACGGCCTGCGTGAAGTCGAAGTTATCTACCGCTCTAAAGCCTGTCT
GATTCAGCGCGACGGTGAGATGGCACAGAGCAAGCAAGAGCTGATCGATAAATTGCTGACC
CGTATTCAGGGCGTGATTCAGGCGCGCGAATCGAAATACATCATGATGCACGCGCCAAGTG
AACGCCTGGAAGAGGTTATCGCCCTGCTGCCAGGCGCCGAAAGGCCGACAATTCTGCCGCT
GGCAGGCGAGCAACAGCGCGTGGCGATGCACATGGTCAGCAGCGAAACGTTGTTCTGGGAA
ACCATGGAGAAACTGAAAGCGCTTGGCGCCAGCTCGATTCTGGTACTGCCGATCGAGAAGA
TGATGGAGTGATCTGACGCCTGATGGCGCTGCGCTTATCAGGCCTACGTAATGCGTTGATAT
TTTGGGTTCTGTAGGCCGGATAAGGCGGAACCCTGTGATGGAGTAAAGACCATGAGCTTCAA
TACCCTGATTGACTGGAACAGCGGATCTGGCGGCCGCGCACTTGTAGAGCACACTAGGGATT
TAGAGGGGATTATGGCACGTACAATATAGATAATTAAGCAGTAGCTGAGTCAGTTGAATGA
TCAGAGGTGTAACATGAGTGTGGATGGATTGTGTAGAGTCGTTGTTTAAAATAATGAGTTAA
GAATAATTATACGACTACAGGATACGATGTACTTGTATTGTATCGATACAGTACATACAGTA
CATACGTGTAACATACTCCTAAACTGTTGCATCACCTACAACTCCAACTAGTCGGTCATAATT
CATTAATACGTTTCCCTGGTGTAGTCTAGGCCAAACACTCCTTGGTGAACTTCTTGAGAAAG
ATTCGCTCTCTAGACTCCTTGAATTCTCTAGGCTGGATTTCGGCCCAAAAAGAACCCACAAA
CGGCGCAATACTCTTCTTGTAGCCGGAATAGTTGTCTCGAATGACAATATCCGGGTTGGCAG
ACCGCTGCTGATGCTCCTTGACGATGATACTCTCAAAGTGGTCCACGGTAGACATGACAATA
AGAGCCGACGAGGTTTTGCTCTTGTAGTTGGCGATGAGCGTTTTAAGTGCCCCAGAGTTCTC
CACCACCTTGTGGATCAGCTGATGCTTTTGTTCCTCGCTGAGTCCTTCCGAGGCCAGGGGGAT
AGCCAAGCAGGTGAGAATAGCGCCTGTGTCTCGTTTAGAAGAGGTGCTGTTTGTATCATCCG
CATGCTTGTTGATGAACTGAACTAAATTCAGTAGCGTGCTCCACAGCTCATTCCAGTGGTAA
TTTGAGGGAGTGTCCCTGTAAACCATGAGTGTTGCTTCAGTGACGACCAGAGCGAGATCATA
AATGTCTGGGAGAGGTTTCTTCATATTGTATCTGAGACAGCAAAGAAGTGCGTCCAAAAGGC
CGGTTCCAAAGGTGTAGGCCTCTGTTTCTGGAAGGGGGGGTTTCCGTTGCTTGGCGTTGATTT
TAATAGATGCTTTTGTGTTGAACGACTGGTGCAATTCGGGCAGAAGAAGCTGCAGAATGATT
AGAGCCACCTTACTGTACATTTCAGCTCGTGGGTTTTTGTTTTGGTTGGAAAAGAGGTACGA
ACAGAGAGAGAGAAAGGCTGCCAGGGCAGGCTCGGTGTCATAGTTCTTGGTACGATGTCCT
TTATCGGTAATGAGTGTCTTTGCAAACAACTTGTTCTTCTGCACGAATTCCAAAGTGGGGGC
CGGCC,

SEQ NO 10

Construct 162. *cbh1-cbh2-eg2* (Length: 7780 bp). Cloning vector: pUC57.

Sequence:

SalI site (GTCGAC) and PmlI site (CACGTG) at the 5' end. KpnI site (GGTACC) at the 3' end.

GTCGACCACGTGAGAGACCGGGTTGGCGGCGCATTTGTGTCCCAAAAAACAGCCCCAATTG
CCCCAATTGACCCCAAATTGACCCAGTAGCGGGCCCAACCCCGGCGAGAGCCCCCTTCTCCC
CACATATCAAACCTCCCCCGGTTCCCACACTTGCCGTTAAGGGCGTAGGGTACTGCAGTCTG

GAATCTACGCTTGTTCAGACTTTGTACTAGTTTCTTTGTCTGGCCATCCGGGTAACCCATGCC
GGACGCAAAATAGACTACTGAAAATTTTTTTGCTTTGTGGTTGGGACTTTAGCCAAGGGTAT
AAAAGACCACCGTCCCCGAATTACCTTTCCTCTTCTTTTCTCTCTCTCCTTGTCAACTCACACC
CGAAATCGTTAAGCATTTCCTTCTGAGTATAAGAATCATTCAAAATGGTGAGTTTCAGAGGC
AGCAGCAATTGCCACGGGCTTTGAGCACACGGCCGGGTGTGGTCCCATTCCCATCGACACAA
GACGCCACGTCATCCGACCAGCACTTTTTGCAGTACTAACCGCAGAAGCTCGCTACCGCCTT
TACTATTCTCACGGCCGTTCTGGCCCAGCAGGCAGGGACCGCTACCGCCGAGAACCATCCTC
CCCTCACCTGGCAGGAGTGCACCGCACCCGGATCCTGCACCACGCAGAACGGCGCTGTCGTG
CTGGACGCAAATTGGCGATGGGTTCACGATGTGAACGGGTACACTAACTGCTATACAGGCA
ACACATGGGATCCTACCTACTGCCCCGACGACGAAACATGTGCCCAGAACTGTGCCCTTGAC
GGAGCTGACTACGAGGGAACCTACGGGGTCACCTCGTCCGGGAGCAGTCTCAAGCTCAACT
TTGTGACCGGTTCAAACGTCGGTTCACGGCTCTATCTGCTCCAGGACGACTCGACCTACCAG
ATCTTCAAACTGTTGAACCGAGAGTTCTCGTTTGACGTTGATGTTTCGAACTTGCCTTGCGGA
CTTAATGGTGCTCTGTACTTTGTTGCAATGGATGCTGACGGCGGAGTCTCTAAGTACCCCAA
CAACAAGGCTGGTGCCAAGTACGGTACGGGCTATTGTGACAGTCAGTGCCCTAGAGATCTA
AAATTCATTGATGGCGAGGCCAACGTCGAGGGCTGGCAACCGAGCAGCAATAACGCCAATA
CTGGAATCGGCGACCACGGCTCCTGTTGCGCGGAGATGGACGTGTGGGAAGCGAACTCCAT
TAGTAATGCTGTAACACCCCATCCGTGCGACACTCCGGGACAGACGATGTGTTCCGGTGACG
ATTGTGGCGGCACCTACTCCAACGATCGATACGCGGGTACATGCGATCCCGACGGCTGCGAT
TTCAACCCATACCGGATGGGTAATACATCCTTCTATGGACCAGGTAAGATTATCGACACTAC
GAAGCCTTTCACCGTGGTCACGCAGTTTTTGACAGATGACGGCACTGATACCGGAACCCTTT
CGGAAATCAAGCGCTTTTACATCCAGAACTCTAATGTTATTCCCCAACCCAACAGTGACATT
TCCGGAGTTACTGGAAACTCTATCACTACAGAGTTTTGTACAGCCCAAAAGCAGGCCTTCGG
GGACACCGACGACTTTTCTCAGCACGGAGGCCTGGCCAAAATGGGAGCTGCCATGCAACAG
GGAATGGTGCTTGTCATGTCTCTGTGGGATGACTACGCCGCTCAAATGCTGTGGCTGGACTC
TGACTACCCCACTGATGCCGATCCAACTACCCCTGGCATCGCCAGAGGTACTTGTCCCACCG
ACAGCGGCGTGCCGTCCGACGTAGAGTCCCAGTCGCCCAACAGCTATGTGACGTACTCGAAC
ATTAAGTTCGGCCCTATCAACTCTACGTTCACCGCATCTAACCCACCTGGTGGTAACCGAGG
TACTACGACCACTCGACGTCCTGCTACTACCACTGGTTCGTCACCCGGACCCACCCAGTCTC
ACTACGGACAGTGTGGCGGAATTGGATACTCTGGTCCCACCGTGTGTGCGTCTGGAACTACC
TGTCAGGTCCTCAACCCTTACTACTCCCAATGCCTGTAATAGGCAATTAACAGATAGTTTGCC
GGTGATAATTCTCTTAACCTCCCACACTCCTTTGACATAACGATTTATGTAACGAAACTGAA
ATTTGACCAGATATTGTTGTAAATAGAAAATCTGGCTTGTAGGTGGCAAAATGCGGCGTCTT
TGTTCATCAATTCCCTCTGTGACTACTCGTCATCCCTTTATGTTCGACTGTCGTATTTCTTATT
TTCCATACATATGCAAGTGAGATGCCCGTGTCCGAATTCGACGCAGTAGGATGTCCTGCACG
GGTCTTTTTGTGGGGTGTGGAGAAAGGGGTGCTTGGAGATGGAAGCCGGTAGAACCGGGCT
GCTTGTGCTTGGAGATGGAAGCCGGTAGAACCGGGCTGCTTGGGGGGATTTGGGGCCGCTG
GGCTCCAAAGAGGGGTAGGCATTTCGTTGGGGTTACGTAATTGCGGCATTTGGGTCCTGCGC
GCATGTCCCATTGGTCAGAATTAGTCCGGATAGGAGACTTATCAGCCAATCACAGCGCCGGA
TCCACCTGTAGGTTGGGTTGGGTGGGAGCACCCCTCCACAGAGTAGAGTCAAACAGCAGCA
GCAACATGATAGTTGGGGGTGTGCGTGTTAAAGGAAAAAAAAGAAGCTTGGGTTATATTCC
CGCTCTATTTAGAGGTTGCGGGATAGACGCCGACGGAGGGCAATGGCGCCATGGAACCTTG
CGGATATCGATACGCCGCGGCGGACTGCGTCCGAACCAGCTCCAGCAGCGTTTTTCCGGGC
CATTGAGCCGACTGCGACCCCGCCAACGTGTCTTGGCCCACGCACTCATGTCATGTTGGTGT

TGGGAGGCCACTTTTTAAGTAGCACAAGGCACCTAGCTCGCAGCAAGGTGTCCGAACCAAA
GAAGCGGCTGCAGTGGTGCAAACGGGGCGGAAACGGCGGGAAAAAGCCACGGGGGCACGA
ATTGAGGCACGCCCTCGAATTTGAGACGAGTCACGGCCCCATTCGCCCGCGCAATGGCTCGC
CAACGCCCGGTCTTTTGCACCACATCAGGTTACCCCAAGCCAAACCTTTGTGTTAAAAAGCT
TAACATATTATACCGAACGTAGGTTTGGGCGGGCTTGCTCCGTCTGTCCAAGGCAACATTTA
TATAAGGGTCTGCATCGCCGGCTCAATTGAATCTTTTTTCTTCTTCTCTTCTCTATATTCATTC
TTGAATTAAACACACATCAACATGAAGCTCGCTACCGCCTTTACTATTCTCACGGCCGTTCTG
GCCCAGGCGTGCAGTAGTGTTTGGGGCCAGTGCGGTGGTCAAAACTGGTCTGGCCCGACTTG
CTGTGCTTCTGGTTCGACCTGTGTTTACTCGAACGATTACTACTCTCAATGCCTGCCGGGAGC
TGCCAGCTCCAGTTCATCCACGAGAGCCGCGTCTACAACATCACGAGTGTCCCCTACGACCT
CTCGCTCATCGAGCGCAACTCCTCCCCCTGGGTCTACCACAACCCGGGTCCCACCTGTGGGA
TCGGGAACTGCCACGTACTCCGGCAATCCTTTTGTGGGCGTGACACCTTGGGCAAACGCCTA
CTACGCTAGTGAAGTGTCGTCGCTGGCCATTCCCTCTCTCACTGGAGCTATGGCAACGGCTG
CCGCTGCCGTCGCAAAGGTCCCCTCCTTCATGTGGCTGGACACTCTTGATAAGACCCCACTC
ATGGAGCAAACTCTGGCAGACATTCGAACTGCGAACAAAAACGGTGGTAACTACGCCGGAC
AGTTCGTGGTCTATGACCTTCCCGACAGAGACTGCGCTGCCCTGGCCTCTAACGGCGAATAT
TCTATTGCGGATGGAGGGGTCGCCAAGTACAAAAACTATATTGACACGATCCGGCAGATCGT
CGTTGAGTACTCTGACATCCGTACCCTCCTCGTCATCGAGCCCGATTCCCTTGCCAACTTGGT
TACCAACCTGGGTACACCTAAGTGTGCAAACGCCCAGTCTGCTTACCTGGAGTGTATTAACT
ATGCTGTTACCCAACTGAACCTCCCCAATGTCGCCATGTACCTCGATGCTGGACACGCCGGT
TGGCTGGGGTGGCCTGCTAATCAGGACCCCGCTGCTCAGCTTTTCGCTAATGTATACAAAAA
CGCTTCCTCCCCCAGGGCCCTTCGAGGACTCGCCACTAACGTTGCCAACTACAACGGTTGGA
ACATTACCTCTCCCCCATCCTACACCCAGGGTAACGCAGTGTATAACGAGAAGCTGTACATC
CATGCTATCGGACCCCTGCTCGCCAATCATGGATGGAGCAACGCCTTTTTCATCACCGACCA
GGGCCGATCGGGCAAGCAGCCCACCGGCCAGCAGCAGTGGGGCGATTGGTGTAATGTGATC
GGAACCGGTTTCGGTATTCGTCCATCTGCCAATACAGGCGATTCCCTGCTCGACTCATTTGTG
TGGGTCAAGCCTGGCGGAGAGTGTGACGGCACCTCGGACAGCTCCGCTCCGCGATTCGATA
GCCACTGCGCACTGCCGGACGCTTTGCAGCCCGCTCCTCAGGCCGGAGCGTGGTTCCAAGCC
TACTTTGTGCAGCTATTGACTAACGCCAACCCCTCGTTTCTATGATAAGCTATTTATCACTCT
TTACAACTTCTACCTCAACTATCTACTTTAATAAATGAATATCGTTTATTCTCTATGATTACTG
TATATGCGTTCCTCTAAGACAAATCGAAACCAGCATGCGATCGAATGGCATACAAAAGTTTC
TTCCGAAGTTGATCAATGTCCTGATAGTCAGGCAGCTTGAGAAGATTGACACAGGTGGAGGC
CGTAGGGAACCGATCAACCTGTCTACCAGCGTTACGAATGGCAAATGACGGGTTCAAAGCC
TTGAATCCTTGCAATGGTGCCTTGGATACTGATGTCACAAACTTAAGAAGCAGCCGCTTGTC
CTCTTCCTCGAAACTGAGTTTGGCGCCCGTTTTTTCGAGCCCCACACGTTTCGGTGAGTATGA
GCGGCGGCAGATTCGAGCGTTTCCGGTTTCCGCGGCTGGACGAGAGCCCATGATGGGGGCTC
CCACCACCAGCAATCAGGGCCCTGATTACACACCCACCTGTAATGTCATGCTGTTCATCGTG
GTTAATGCTGCTGTGTGCTGTGTGTGTGTGTTGTTTGGCGCTCATTGTTGCGTTATGCAGCGT
ACACCACAATATTGGAAGCTTATTAGCCTTTCTATTTTTTCGTTTGCAAGGCTTAACAACATT
GCTGTGGAGAGGGATGGGGATATGGAGGCCGCTGGAGGGAGTCGGAGAGGCGTTTTGGAGC
GGCTTGGCCTGGCGCCCAGCTCGCGAAACGCACCTAGGACCCTTTGGCACGCCGAAATGTGC
CACTTTTCAGTCTAGTAACGCCTTACCTACGTCATTCCATGCATGCATGTTTGCGCCTTTTTTC
CCTTGCCCTTGATCGCCACACAGTACAGTGCACTGTACAGTGGAGGTTTTGGGGGGGTCTTA
GATGGGAGCTAAAAGCGGCCTAGCGGTACACTAGTGGGATTGTATGGAGTGGCATGGAGCC

TAGGTGGAGCCTGACAGGACGCACGACCGGCTAGCCCGTGACAGACGATGGGTGGCTCCTG
TTGTCCACCGCGTACAAATGTTTGGGCCAAAGTCTTGTCAGCCTTGCTTGCGAACCTAATTCC
CAATTTTGTCACTTCGCACCCCCATTGATCGAGCCCTAACCCCTGCCCATCAGGCAATCCAAT
TAAGCTCGCATTGTCTGCCTTGTTTAGTTTGGCTCCTGCCCGTTTCGGCGTCCACTTGCACAA
ACACAAACAAGCATTATATATAAGGCTCGTCTCTCCCTCCCAACCACACTCACTTTTTTGCCC
GTCTTCCCTTGCTAACACAAAAGTCAAGAACACAAACAACCACCCCAACCCCCTTACACACA
AGACATATCTACAGCAATGAAGCTCGCTACCGCCTTTACTATTCTCACGGCCGTTCTGGCCC
AACAGACGGTGTGGGGCCAATGTGGCGGCATTGGCTGGTCGGGTCCGACCAACTGTGCTCCT
GGCTCGGCCTGTTCGACTCTGAACCCCTACTACGCTCAGTGTATCCCCGGAGCGACCACCAT
CACCACGTCTACTCGACCCCCTTCAGGACCCACTACCACCACGCGAGCGACCTCCACCTCCA
GCTCTACTCCCCCTACATCCTCTGGTGTTCGATTTGCTGGCGTGAACATCGCCGGTTTCGACT
TCGGATGCACGACTGACGGTACATGCGTGACATCCAAGGTCTATCCCCCGTTGAAAAACTTC
ACCGGGTCTAACAATTATCCTGACGGCATTGGTCAGATGCAACACTTTGTCAACGAGGATGG
CATGACCATCTTCCGACTCCCTGTCGGCTGGCAGTATCTAGTGAACAATAACCTGGGCGGAA
ACCTTGACAGCACAAGCATCTCCAAGTACGATCAATTGGTGCAGGGATGCCTCAGTTTGGGT
GCTTACTGCATCGTTGACATTCACAACTACGCTAGGTGGAACGGGGGAATCATCGGACAGG
GTGGCCCCACTAACGCCCAGTTCACAAGTCTGTGGTCCCAGCTCGCCTCTAAGTACGCATCT
CAGTCCCGGGTGTGGTTTGGAATCATGAATGAGCCCCATGACGTCAACATTAACACTTGGGC
TGCCACCGTACAGGAAGTCGTTACCGCCATTCGTAACGCCGGAGCCACCTCGCAGTTCATCT
CGCTGCCTGGAAATGACTGGCAGTCTGCTGGTGCTTTCATTTCCGATGGATCAGCAGCCGCT
CTCAGCCAAGTCACGAACCCTGATGGATCTACAACCAACCTGATTTTCGACGTGCATAAGTA
CCTGGACTCGGATAACAGCGGAACCCACGCGGAGTGTACCACTAACAACATTGACGGTGCC
TTTTCTCCTCTCGCAACGTGGCTCAGACAGAACAACAGACAGGCCATTCTGACTGAAACTGG
TGGCGGCAACGTTCAATCATGTATCCAGGACATGTGCCAGCAGATTCAGTACCTGAATCAGA
ATAGTGACGTGTACCTTGGCTACGTGGGTTGGGGAGCAGGGTCCTTTGATTCTACCTACGTC
CTGACCGAGACTCCGACATCCTCCGGCAACTCGTGGACTGATACATCTCTTGTTTCGTCATGC
CTTGCCCGCAAGTGATAAAGCTTTGCGGCGAAACTCGATTCTCACCCTCGATAACTCGACTC
ACCCCCCTTAACTTAAAATTCACTTACGACAAACAAACGTCTGATACCGACTACCCCCTCGA
CTTCTCGCAATCTCGACTTTCAATCAGAGGACCTCAAACAACCACTTTTTTCTTACGATTCTA
ATTATTTACCCATTCATTAATTTCCCGTGCGCTCGTCCAGCAATGTCCGAGAGCATCCTGCCT
GTGTCTCTGGGCCCATCCATTTAATTTGGGTCCATCCTCCCGGGCAGATTCCACAGTCCAGTT
GTCGCCGACTGGATGGTTAGTAGATCCGCCTTTTTTAGTTTGAACATGTTGCGAGTTGATACC
TGAACATCAGAGTTTTAGCTTCTTGTACTAATATACATTCCTTTTAGTGAGGTCCTGTACACT
GTACACTGTATTATATTGTAGCGTTGTATTTTTGATCCCTTGGTTTGTTCGTTATACGGTGTAC
AAGTATGTACGATCGGTACC,

SEQ NO 11

Construct 163 (length: 14064 bp) for Snf1up-*cbh1-cbh2-eg2*-HisG-*URA3*-HisG-Snf1down; Cloning vector: pUC57-Simple.

AscI site (GGCGCGCC) at the 5’ end; FseI site (GGCCGGCC) at the 3’ end.

GGCGCGCCTGATTACACCCTGGAACTGTTGGGTGGAGACAAGCCAGATATTCGGGAGATCC
AGCTGAATCTGAACGGGTTTCTGGAGGAGAACACAGCCAAGTTCTGCAAGGAGCTGTGGGA
GCTGCTGGTGGCTGCCCAGAAAGACAAGGACGGCATTCCGCCGCAGTTGATTGCCATCAAG
AAGGAGCAGATGGAGCAGGAGCGGGTGAAGCGGGAAATCAAGATTGAGAGTTGGAGTGGT
GGGGGCGGTGGTGGAAGAGACAGAGGTGATAGAAGGGAAAGAAACGACAGAAGAGACGC
AAAAGGAAGAAACAGAAGAGATGGAGATAGGAGGAATGGCGATCGAAGAGACAGAGACA
GAGATAGAGATAGAGACGACAGGTCAAGTCGTTCAAGTCGGTACAACCGATCAAGATCAAG
ATCTCCGACTGTCAAAAAGGAAACAGATGAATATGGCCGGGACCGGAAGGACTAACTGCAT
TATATATAAATATAGATTCTTGATTAGTGATCAAAGCATGAGATACTGTCAAGGCAAGCAAG
CCTTACAATATCTGTTGGCTTAGTTAAATCTTTTTTGAGATGTAATAATTGGGGAACCTCTGT
TAGATGAACAGTTGCGGCTTACTCATCTTAGACATGGTCCAGTTTAGGCGTAATTAATTATC
AGTTTTAATGCGCCTCTACGTAATAAGCGTTGCCACAAGTTACAGTAGAGATGATACCATTG
CTACAGGACCGTATAGCGTATATAATGACGAGGCTAATTGATGATGATCGACAGCGAAGAC
ATATCTACGTCAATCTGACGCTTCATTTTGTGCTTCTTGTTCGGATTTGTGTTTCTCCCAGTGC
ACCCAGTTTTTTGCTGACCATTTTTTGCTTACCAGTTTTTGCTCGCGCGTTTTCTCGATTGAAC
TCTGCTGCGAGGGTGGTGTAAGACACACTCCCTGTAGAACATGCATTTCGAGGTGTGACATT
TCCATCTCTGTTAAATCAAACGAGTGACACAAACGGAATAAATCTACATATACGTCGAACCC
TCTTCTTCTCTTCTCTCTCTTCCTTAATAGACACCCTCGCTATCGACTCTGCTCCTCCTTATCA
CACCATCAAAGTTTAGGGTGTGTCCCAACACCACCTCCACACCACACTCTATTATCACCACC
TCCACGACTACATACCACTCCTTCCACCACCTCACAACACGTGAGAGACCGGGTTGGCGGCG
CATTTGTGTCCCAAAAAACAGCCCCAATTGCCCCAATTGACCCCAAATTGACCCAGTAGCGG
GCCCAACCCCGGCGAGAGCCCCCTTCTCCCCACATATCAAACCTCCCCCGGTTCCCACACTT
GCCGTTAAGGGCGTAGGGTACTGCAGTCTGGAATCTACGCTTGTTCAGACTTTGTACTAGTTT
CTTTGTCTGGCCATCCGGGTAACCCATGCCGGACGCAAAATAGACTACTGAAAATTTTTTTG
CTTTGTGGTTGGGACTTTAGCCAAGGGTATAAAAGACCACCGTCCCCGAATTACCTTTCCTCT
TCTTTTCTCTCTCTCCTTGTCAACTCACACCCGAAATCGTTAAGCATTTCCTTCTGAGTATAAG
AATCATTCAAAATGGTGAGTTTCAGAGGCAGCAGCAATTGCCACGGGCTTTGAGCACACGG
CCGGGTGTGGTCCCATTCCCATCGACACAAGACGCCACGTCATCCGACCAGCACTTTTTGCA
GTACTAACCGCAGAAGCTCGCTACCGCCTTTACTATTCTCACGGCCGTTCTGGCCCAGCAGG
CAGGGACCGCTACCGCCGAGAACCATCCTCCCCTCACCTGGCAGGAGTGCACCGCACCCGG
ATCCTGCACCACGCAGAACGGCGCTGTCGTGCTGGACGCAAATTGGCGATGGGTTCACGATG
TGAACGGGTACACTAACTGCTATACAGGCAACACATGGGATCCTACCTACTGCCCCGACGAC
GAAACATGTGCCCAGAACTGTGCCCTTGACGGAGCTGACTACGAGGGAACCTACGGGGTCA
CCTCGTCCGGGAGCAGTCTCAAGCTCAACTTTGTGACCGGTTCAAACGTCGGTTCACGGCTC
TATCTGCTCCAGGACGACTCGACCTACCAGATCTTCAAACTGTTGAACCGAGAGTTCTCGTTT
GACGTTGATGTTTCGAACTTGCCTTGCGGACTTAATGGTGCTCTGTACTTTGTTGCAATGGAT
GCTGACGGCGGAGTCTCTAAGTACCCCAACAACAAGGCTGGTGCCAAGTACGGTACGGGCT
ATTGTGACAGTCAGTGCCCTAGAGATCTAAAATTCATTGATGGCGAGGCCAACGTCGAGGGC
TGGCAACCGAGCAGCAATAACGCCAATACTGGAATCGGCGACCACGGCTCCTGTTGCGCGG
AGATGGACGTGTGGGAAGCGAACTCCATTAGTAATGCTGTAACACCCCATCCGTGCGACACT
CCGGGACAGACGATGTGTTCCGGTGACGATTGTGGCGGCACCTACTCCAACGATCGATACGC
GGGTACATGCGATCCCGACGGCTGCGATTTCAACCCATACCGGATGGGTAATACATCCTTCT
ATGGACCAGGTAAGATTATCGACACTACGAAGCCTTTCACCGTGGTCACGCAGTTTTTGACA
GATGACGGCACTGATACCGGAACCCTTTCGGAAATCAAGCGCTTTTACATCCAGAACTCTAA

TGTTATTCCCCAACCCAACAGTGACATTTCCGGAGTTACTGGAAACTCTATCACTACAGAGT
TTTGTACAGCCCAAAAGCAGGCCTTCGGGGACACCGACGACTTTTCTCAGCACGGAGGCCTG
GCCAAAATGGGAGCTGCCATGCAACAGGGAATGGTGCTTGTCATGTCTCTGTGGGATGACTA
CGCCGCTCAAATGCTGTGGCTGGACTCTGACTACCCCACTGATGCCGATCCAACTACCCCTG
GCATCGCCAGAGGTACTTGTCCCACCGACAGCGGCGTGCCGTCCGACGTAGAGTCCCAGTCG
CCCAACAGCTATGTGACGTACTCGAACATTAAGTTCGGCCCTATCAACTCTACGTTCACCGC
ATCTAACCCACCTGGTGGTAACCGAGGTACTACGACCACTCGACGTCCTGCTACTACCACTG
GTTCGTCACCCGGACCCACCCAGTCTCACTACGGACAGTGTGGCGGAATTGGATACTCTGGT
CCCACCGTGTGTGCGTCTGGAACTACCTGTCAGGTCCTCAACCCTTACTACTCCCAATGCCTG
TAATAGGCAATTAACAGATAGTTTGCCGGTGATAATTCTCTTAACCTCCCACACTCCTTTGAC
ATAACGATTTATGTAACGAAACTGAAATTTGACCAGATATTGTTGTAAATAGAAAATCTGGC
TTGTAGGTGGCAAAATGCGGCGTCTTTGTTCATCAATTCCCTCTGTGACTACTCGTCATCCCT
TTATGTTCGACTGTCGTATTTCTTATTTTCCATACATATGCAAGTGAGATGCCCGTGTCCGAA
TTCGACGCAGTAGGATGTCCTGCACGGGTCTTTTTGTGGGGTGTGGAGAAAGGGGTGCTTGG
AGATGGAAGCCGGTAGAACCGGGCTGCTTGTGCTTGGAGATGGAAGCCGGTAGAACCGGGC
TGCTTGGGGGGATTTGGGGCCGCTGGGCTCCAAAGAGGGGTAGGCATTTCGTTGGGGTTACG
TAATTGCGGCATTTGGGTCCTGCGCGCATGTCCCATTGGTCAGAATTAGTCCGGATAGGAGA
CTTATCAGCCAATCACAGCGCCGGATCCACCTGTAGGTTGGGTTGGGTGGGAGCACCCCTCC
ACAGAGTAGAGTCAAACAGCAGCAGCAACATGATAGTTGGGGGTGTGCGTGTTAAAGGAAA
AAAAAGAAGCTTGGGTTATATTCCCGCTCTATTTAGAGGTTGCGGGATAGACGCCGACGGAG
GGCAATGGCGCCATGGAACCTTGCGGATATCGATACGCCGCGGCGGACTGCGTCCGAACCA
GCTCCAGCAGCGTTTTTTCCGGGCCATTGAGCCGACTGCGACCCCGCCAACGTGTCTTGGCC
CACGCACTCATGTCATGTTGGTGTTGGGAGGCCACTTTTTAAGTAGCACAAGGCACCTAGCT
CGCAGCAAGGTGTCCGAACCAAAGAAGCGGCTGCAGTGGTGCAAACGGGGCGGAAACGGC
GGGAAAAAGCCACGGGGGCACGAATTGAGGCACGCCCTCGAATTTGAGACGAGTCACGGCC
CCATTCGCCCGCGCAATGGCTCGCCAACGCCCGGTCTTTTGCACCACATCAGGTTACCCCAA
GCCAAACCTTTGTGTTAAAAAGCTTAACATATTATACCGAACGTAGGTTTGGGCGGGCTTGC
TCCGTCTGTCCAAGGCAACATTTATATAAGGGTCTGCATCGCCGGCTCAATTGAATCTTTTTT
CTTCTTCTCTTCTCTATATTCATTCTTGAATTAAACACACATCAACATGAAGCTCGCTACCGC
CTTTACTATTCTCACGGCCGTTCTGGCCCAGGCGTGCAGTAGTGTTTGGGGCCAGTGCGGTG
GTCAAAACTGGTCTGGCCCGACTTGCTGTGCTTCTGGTTCGACCTGTGTTTACTCGAACGATT
ACTACTCTCAATGCCTGCCGGGAGCTGCCAGCTCCAGTTCATCCACGAGAGCCGCGTCTACA
ACATCACGAGTGTCCCCTACGACCTCTCGCTCATCGAGCGCAACTCCTCCCCCTGGGTCTACC
ACAACCCGGGTCCCACCTGTGGGATCGGGAACTGCCACGTACTCCGGCAATCCTTTTGTGGG
CGTGACACCTTGGGCAAACGCCTACTACGCTAGTGAAGTGTCGTCGCTGGCCATTCCCTCTC
TCACTGGAGCTATGGCAACGGCTGCCGCTGCCGTCGCAAAGGTCCCCTCCTTCATGTGGCTG
GACACTCTTGATAAGACCCCACTCATGGAGCAAACTCTGGCAGACATTCGAACTGCGAACA
AAAACGGTGGTAACTACGCCGGACAGTTCGTGGTCTATGACCTTCCCGACAGAGACTGCGCT
GCCCTGGCCTCTAACGGCGAATATTCTATTGCGGATGGAGGGGTCGCCAAGTACAAAAACTA
TATTGACACGATCCGGCAGATCGTCGTTGAGTACTCTGACATCCGTACCCTCCTCGTCATCGA
GCCCGATTCCCTTGCCAACTTGGTTACCAACCTGGGTACACCTAAGTGTGCAAACGCCCAGT
CTGCTTACCTGGAGTGTATTAACTATGCTGTTACCCAACTGAACCTCCCCAATGTCGCCATGT
ACCTCGATGCTGGACACGCCGGTTGGCTGGGGTGGCCTGCTAATCAGGACCCCGCTGCTCAG
CTTTTCGCTAATGTATACAAAAACGCTTCCTCCCCCAGGGCCCTTCGAGGACTCGCCACTAA

CGTTGCCAACTACAACGGTTGGAACATTACCTCTCCCCCATCCTACACCCAGGGTAACGCAG
TGTATAACGAGAAGCTGTACATCCATGCTATCGGACCCCTGCTCGCCAATCATGGATGGAGC
AACGCCTTTTTCATCACCGACCAGGGCCGATCGGGCAAGCAGCCCACCGGCCAGCAGCAGT
GGGGCGATTGGTGTAATGTGATCGGAACCGGTTTCGGTATTCGTCCATCTGCCAATACAGGC
GATTCCCTGCTCGACTCATTTGTGTGGGTCAAGCCTGGCGGAGAGTGTGACGGCACCTCGGA
CAGCTCCGCTCCGCGATTCGATAGCCACTGCGCACTGCCGGACGCTTTGCAGCCCGCTCCTC
AGGCCGGAGCGTGGTTCCAAGCCTACTTTGTGCAGCTATTGACTAACGCCAACCCCTCGTTT
CTATGATAAGCTATTTATCACTCTTTACAACTTCTACCTCAACTATCTACTTTAATAAATGAA
TATCGTTTATTCTCTATGATTACTGTATATGCGTTCCTCTAAGACAAATCGAAACCAGCATGC
GATCGAATGGCATACAAAAGTTTCTTCCGAAGTTGATCAATGTCCTGATAGTCAGGCAGCTT
GAGAAGATTGACACAGGTGGAGGCCGTAGGGAACCGATCAACCTGTCTACCAGCGTTACGA
ATGGCAAATGACGGGTTCAAAGCCTTGAATCCTTGCAATGGTGCCTTGGATACTGATGTCAC
AAACTTAAGAAGCAGCCGCTTGTCCTCTTCCTCGAAACTGAGTTTGGCGCCCGTTTTTTCGAG
CCCCACACGTTTCGGTGAGTATGAGCGGCGGCAGATTCGAGCGTTTCCGGTTTCCGCGGCTG
GACGAGAGCCCATGATGGGGGCTCCCACCACCAGCAATCAGGGCCCTGATTACACACCCAC
CTGTAATGTCATGCTGTTCATCGTGGTTAATGCTGCTGTGTGCTGTGTGTGTGTGTTGTTTGG
CGCTCATTGTTGCGTTATGCAGCGTACACCACAATATTGGAAGCTTATTAGCCTTTCTATTTT
TTCGTTTGCAAGGCTTAACAACATTGCTGTGGAGAGGGATGGGGATATGGAGGCCGCTGGA
GGGAGTCGGAGAGGCGTTTTGGAGCGGCTTGGCCTGGCGCCCAGCTCGCGAAACGCACCTA
GGACCCTTTGGCACGCCGAAATGTGCCACTTTTCAGTCTAGTAACGCCTTACCTACGTCATTC
CATGCATGCATGTTTGCGCCTTTTTTCCCTTGCCCTTGATCGCCACACAGTACAGTGCACTGT
ACAGTGGAGGTTTTGGGGGGGTCTTAGATGGGAGCTAAAAGCGGCCTAGCGGTACACTAGT
GGGATTGTATGGAGTGGCATGGAGCCTAGGTGGAGCCTGACAGGACGCACGACCGGCTAGC
CCGTGACAGACGATGGGTGGCTCCTGTTGTCCACCGCGTACAAATGTTTGGGCCAAAGTCTT
GTCAGCCTTGCTTGCGAACCTAATTCCCAATTTTGTCACTTCGCACCCCCATTGATCGAGCCC
TAACCCCTGCCCATCAGGCAATCCAATTAAGCTCGCATTGTCTGCCTTGTTTAGTTTGGCTCC
TGCCCGTTTCGGCGTCCACTTGCACAAACACAAACAAGCATTATATATAAGGCTCGTCTCTC
CCTCCCAACCACACTCACTTTTTTGCCCGTCTTCCCTTGCTAACACAAAAGTCAAGAACACAA
ACAACCACCCCAACCCCCTTACACACAAGACATATCTACAGCAATGAAGCTCGCTACCGCCT
TTACTATTCTCACGGCCGTTCTGGCCCAACAGACGGTGTGGGGCCAATGTGGCGGCATTGGC
TGGTCGGGTCCGACCAACTGTGCTCCTGGCTCGGCCTGTTCGACTCTGAACCCCTACTACGCT
CAGTGTATCCCCGGAGCGACCACCATCACCACGTCTACTCGACCCCCTTCAGGACCCACTAC
CACCACGCGAGCGACCTCCACCTCCAGCTCTACTCCCCCTACATCCTCTGGTGTTCGATTTGC
TGGCGTGAACATCGCCGGTTTCGACTTCGGATGCACGACTGACGGTACATGCGTGACATCCA
AGGTCTATCCCCCGTTGAAAAACTTCACCGGGTCTAACAATTATCCTGACGGCATTGGTCAG
ATGCAACACTTTGTCAACGAGGATGGCATGACCATCTTCCGACTCCCTGTCGGCTGGCAGTA
TCTAGTGAACAATAACCTGGGCGGAAACCTTGACAGCACAAGCATCTCCAAGTACGATCAA
TTGGTGCAGGGATGCCTCAGTTTGGGTGCTTACTGCATCGTTGACATTCACAACTACGCTAG
GTGGAACGGGGGAATCATCGGACAGGGTGGCCCCACTAACGCCCAGTTCACAAGTCTGTGG
TCCCAGCTCGCCTCTAAGTACGCATCTCAGTCCCGGGTGTGGTTTGGAATCATGAATGAGCC
CCATGACGTCAACATTAACACTTGGGCTGCCACCGTACAGGAAGTCGTTACCGCCATTCGTA
ACGCCGGAGCCACCTCGCAGTTCATCTCGCTGCCTGGAAATGACTGGCAGTCTGCTGGTGCT
TTCATTTCCGATGGATCAGCAGCCGCTCTCAGCCAAGTCACGAACCCTGATGGATCTACAAC
CAACCTGATTTTCGACGTGCATAAGTACCTGGACTCGGATAACAGCGGAACCCACGCGGAGT

GTACCACTAACAACATTGACGGTGCCTTTTCTCCTCTCGCAACGTGGCTCAGACAGAACAAC
AGACAGGCCATTCTGACTGAAACTGGTGGCGGCAACGTTCAATCATGTATCCAGGACATGTG
CCAGCAGATTCAGTACCTGAATCAGAATAGTGACGTGTACCTTGGCTACGTGGGTTGGGGAG
CAGGGTCCTTTGATTCTACCTACGTCCTGACCGAGACTCCGACATCCTCCGGCAACTCGTGG
ACTGATACATCTCTTGTTTCGTCATGCCTTGCCCGCAAGTGATAAAGCTTTGCGGCGAAACTC
GATTCTCACCCTCGATAACTCGACTCACCCCCCTTAACTTAAAATTCACTTACGACAAACAA
ACGTCTGATACCGACTACCCCCTCGACTTCTCGCAATCTCGACTTTCAATCAGAGGACCTCA
AACAACCACTTTTTTCTTACGATTCTAATTATTTACCCATTCATTAATTTCCCGTGCGCTCGTC
CAGCAATGTCCGAGAGCATCCTGCCTGTGTCTCTGGGCCCATCCATTTAATTTGGGTCCATCC
TCCCGGGCAGATTCCACAGTCCAGTTGTCGCCGACTGGATGGTTAGTAGATCCGCCTTTTTTA
GTTTGAACATGTTGCGAGTTGATACCTGAACATCAGAGTTTTAGCTTCTTGTACTAATATACA
TTCCTTTTAGTGAGGTCCTGTACACTGTACACTGTATTATATTGTAGCGTTGTATTTTTGATCC
CTTGGTTTGTTCGTTATACGGTGTACAAGTATGTACGATCGGTACCACGCGTAGATCTTCCAG
TGGTGCATGAACGCATGAGAAAGCCCCCGGAAGATCATCTTCCGGGGGCTTTTTTTTTGGCG
CGCGATACAGACCGGTTCAGACAGGATAAAGAGGAACGCAGAATGTTAGACAACACCCGCT
TACGCATAGCTATTCAGAAATCAGGCCGTTTAAGCGATGATTCACGAGAATTGCTGGCCCGC
TGCGGCATAAAAATTAATTTACACACTCAGCGCCTGATTGCGATGGCGGAAAACATGCCGAT
TGATATCCTGCGCGTGCGTGATGATGACATTCCGGGTCTGGTAATGGATGGCGTGGTCGATC
TCGGTATTATCGGCGAAAACGTGCTGGAAGAAGAGCTACTCAACCGCCGCGCACAGGGCGA
AGATCCACGCTATTTAACCCTGCGCCGTCTTGACTTCGGCGGCTGCCGTTTATCGCTGGCAAC
ACCGGTTGACGAAGCCTGGGACGGCCCGGCCGCGCTGGACGGTAAACGTATCGCTACCTCA
TATCCGCACCTCCTCAAACGCTACCTCGACCAGAAAGGCGTCTCTTTTAAATCGTGTCTGTTA
AATGGTTCTGTCGAAGTCGCGCCGCGCGCGGGGCTGGCCGACGCTATCTGCGATTTGGTCTC
TACCGGCGCGACGCTTGAAGCTAACGGCCTGCGTGAAGTCGAAGTTATCTACCGCTCTAAAG
CCTGTCTGATTCAGCGCGACGGTGAGATGGCACAGAGCAAGCAAGAGCTGATCGATAAATT
GCTGACCCGTATTCAGGGCGTGATTCAGGCGCGCGAATCGAAATACATCATGATGCACGCGC
CAAGTGAACGCCTGGAAGAGGTTATCGCCCTGCTGCCAGGCGCCGAAAGGCCGACAATTCT
GCCGCTGGCAGGCGAGCAACAGCGCGTGGCGATGCACATGGTCAGCAGCGAAACGTTGTTC
TGGGAAACCATGGAGAAACTGAAAGCGCTTGGCGCCAGCTCGATTCTGGTACTGCCGATCG
AGAAGATGATGGAGTGATCTGACGCCTGATGGCGCTGCGCTTATCAGGCCTACGTAATGCGT
TGATATTTTGGGTTCTGTAGGCCGGATAAGGCGGAACCCTGTGATGGAGTAAAGACCATGAG
CTTCAATACCCTGATTGACTGGAACAGCGGATCTGGTCGACGAGTATCTGTCTGACTCGTCA
TTGCCGCCTTTGGAGTACGACTCCAACTATGAGTGTGCTTGGATCACTTTGACGATACATTCT
TCGTTGGAGGCTGTGGGTCTGACAGCTGCGTTTTCGGCGCGGTTGGCCGACAACAATATCAG
CTGCAACGTCATTGCTGGCTTTCATCATGATCACATTTTTGTCGGCAAAGGCGACGCCCAGA
GAGCCATTGACGTTCTTTCTAATTTGGACCGATAGCCGTATAGTCCAGTCTATCTATAAGTTC
AACTAACTCGTAACTATTACCATAACATATACTTCACTGCCCCAGATAAGGTTCCGATAAAA
AGTTCTGCAGACTAAATTTATTTCAGTCTCCTCTTCACCACCAAAATGCCCTCCTACGAAGCT
CGAGCTAACGTCCACAAGTCCGCCTTTGCCGCTCGAGTGCTCAAGCTCGTGGCAGCCAAGAA
AACCAACCTGTGTGCTTCTCTGGATGTTACCACCACCAAGGAGCTCATTGAGCTTGCCGATA
AGGTCGGACCTTATGTGTGCATGATCAAGACCCATATCGACATCATTGACGACTTCACCTAC
GCCGGCACTGTGCTCCCCCTCAAGGAACTTGCTCTTAAGCACGGTTTCTTCCTGTTCGAGGAC
AGAAAGTTCGCAGATATTGGCAACACTGTCAAGCACCAGTACAAGAACGGTGTCTACCGAA
TCGCCGAGTGGTCCGATATCACCAACGCCCACGGTGTACCCGGAACCGGAATCATTGCTGGC

CTGCGAGCTGGTGCCGAGGAAACTGTCTCTGAACAGAAGAAGGAGGACGTCTCTGACTACG
AGAACTCCCAGTACAAGGAGTTCCTGGTCCCCTCTCCCAACGAGAAGCTGGCCAGAGGTCTG
CTCATGCTGGCCGAGCTGTCTTGCAAGGGCTCTCTGGCCACTGGCGAGTACTCCAAGCAGAC
CATTGAGCTTGCCCGATCCGACCCCGAGTTTGTGGTTGGCTTCATTGCCCAGAACCGACCTA
AGGGCGACTCTGAGGACTGGCTTATTCTGACCCCCGGGGTGGGTCTTGACGACAAGGGAGA
CGCTCTCGGACAGCAGTACCGAACTGTTGAGGATGTCATGTCTACCGGAACGGATATCATAA
TTGTCGGCCGAGGTCTGTACGGCCAGAACCGAGATCCTATTGAGGAGGCCAAGCGATACCA
GAAGGCTGGCTGGGAGGCTTACCAGAAGATTAACTGTTAGAGGTTAGACTATGGATATGTC
ATTTAACTGTGTATATAGAGAGCGTGCAAGTATGGAGCGCTTGTTCAGCTTGTATGATGGTC
AGACGACCTGTCTGATCGAGTATGTATGATACTGCACAACCTGTGTATCCGCATGATCTGTC
CAATGGGGCATGTTGTTGTGTTTCTCGATACGGAGATGCTGGGTACAAGTAGCTAATACGAT
TGAACTACTTATACTTATATGAGGCTTGAAGAAAGCTGACTTGTGTATGACTTATTCTCAACT
ACATCCCCAGTCACAATACCACCACTGCACGGATCTTCCAGTGGTGCATGAACGCATGAGAA
AGCCCCCGGAAGATCATCTTCCGGGGGCTTTTTTTTTGGCGCGCGATACAGACCGGTTCAGA
CAGGATAAAGAGGAACGCAGAATGTTAGACAACACCCGCTTACGCATAGCTATTCAGAAAT
CAGGCCGTTTAAGCGATGATTCACGAGAATTGCTGGCCCGCTGCGGCATAAAAATTAATTTA
CACACTCAGCGCCTGATTGCGATGGCGGAAAACATGCCGATTGATATCCTGCGCGTGCGTGA
TGATGACATTCCGGGTCTGGTAATGGATGGCGTGGTCGATCTCGGTATTATCGGCGAAAACG
TGCTGGAAGAAGAGCTACTCAACCGCCGCGCACAGGGCGAAGATCCACGCTATTTAACCCT
GCGCCGTCTTGACTTCGGCGGCTGCCGTTTATCGCTGGCAACACCGGTTGACGAAGCCTGGG
ACGGCCCGGCCGCGCTGGACGGTAAACGTATCGCTACCTCATATCCGCACCTCCTCAAACGC
TACCTCGACCAGAAAGGCGTCTCTTTTAAATCGTGTCTGTTAAATGGTTCTGTCGAAGTCGCG
CCGCGCGCGGGGCTGGCCGACGCTATCTGCGATTTGGTCTCTACCGGCGCGACGCTTGAAGC
TAACGGCCTGCGTGAAGTCGAAGTTATCTACCGCTCTAAAGCCTGTCTGATTCAGCGCGACG
GTGAGATGGCACAGAGCAAGCAAGAGCTGATCGATAAATTGCTGACCCGTATTCAGGGCGT
GATTCAGGCGCGCGAATCGAAATACATCATGATGCACGCGCCAAGTGAACGCCTGGAAGAG
GTTATCGCCCTGCTGCCAGGCGCCGAAAGGCCGACAATTCTGCCGCTGGCAGGCGAGCAAC
AGCGCGTGGCGATGCACATGGTCAGCAGCGAAACGTTGTTCTGGGAAACCATGGAGAAACT
GAAAGCGCTTGGCGCCAGCTCGATTCTGGTACTGCCGATCGAGAAGATGATGGAGTGATCTG
ACGCCTGATGGCGCTGCGCTTATCAGGCCTACGTAATGCGTTGATATTTTGGGTTCTGTAGGC
CGGATAAGGCGGAACCCTGTGATGGAGTAAAGACCATGAGCTTCAATACCCTGATTGACTG
GAACAGCGGATCTGGCGGCCGCGCACTTGTAGAGCACACTAGGGATTTAGAGGGGATTATG
GCACGTACAATATAGATAATTAAGCAGTAGCTGAGTCAGTTGAATGATCAGAGGTGTAACA
TGAGTGTGGATGGATTGTGTAGAGTCGTTGTTTAAAATAATGAGTTAAGAATAATTATACGA
CTACAGGATACGATGTACTTGTATTGTATCGATACAGTACATACAGTACATACGTGTAACAT
ACTCCTAAACTGTTGCATCACCTACAACTCCAACTAGTCGGTCATAATTCATTAATACGTTTC
CCTGGTGTAGTCTAGGCCAAACACTCCTTGGTGAACTTCTTGAGAAAGATTCGCTCTCTAGA
CTCCTTGAATTCTCTAGGCTGGATTTCGGCCCAAAAAGAACCCACAAACGGCGCAATACTCT
TCTTGTAGCCGGAATAGTTGTCTCGAATGACAATATCCGGGTTGGCAGACCGCTGCTGATGC
TCCTTGACGATGATACTCTCAAAGTGGTCCACGGTAGACATGACAATAAGAGCCGACGAGG
TTTTGCTCTTGTAGTTGGCGATGAGCGTTTTAAGTGCCCCAGAGTTCTCCACCACCTTGTGGA
TCAGCTGATGCTTTTGTTCCTCGCTGAGTCCTTCCGAGGCCAGGGGGATAGCCAAGCAGGTG
AGAATAGCGCCTGTGTCTCGTTTAGAAGAGGTGCTGTTTGTATCATCCGCATGCTTGTTGATG
AACTGAACTAAATTCAGTAGCGTGCTCCACAGCTCATTCCAGTGGTAATTTGAGGGAGTGTC

CCTGTAAACCATGAGTGTTGCTTCAGTGACGACCAGAGCGAGATCATAAATGTCTGGGAGA
GGTTTCTTCATATTGTATCTGAGACAGCAAAGAAGTGCGTCCAAAAGGCCGGTTCCAAAGGT
GTAGGCCTCTGTTTCTGGAAGGGGGGGTTTCCGTTGCTTGGCGTTGATTTAATAGATGCTTT
TGTGTTGAACGACTGGTGCAATTCGGGCAGAAGAAGCTGCAGAATGATTAGAGCCACCTTA
CTGTACATTTCAGCTCGTGGGTTTTTGTTTTGGTTGGAAAAGAGGTACGAACAGAGAGAGAG
AAAGGCTGCCAGGGCAGGCTCGGTGTCATAGTTCTTGGTACGATGTCCTTTATCGGTAATGA
GTGTCTTTGCAAACAACTTGTTCTTCTGCACGAATTCCAAAGTGGGGGCCGGCC

References

Alani, E., Cao, L., and Kleckner, N. (1987). A method for gene disruption that allows repeated use of URA3 selection in the construction of multiply disrupted yeast strains. *Genetics* 116**,** 541-545.

Tai, M., and Stephanopoulos, G. (2013). Engineering the push and pull of lipid biosynthesis in oleaginous yeast *Yarrowia lipolytica* for biofuel production. *Metabolic Engineering* 15**,** 1-9.

Voth, W.P., Richards, J.D., Shaw, J.M., and Stillman, D.J. (2001). Yeast vectors for integration at the HO locus. *Nucleic Acids Research* 29**,** e59-e59.

Wei, H., Wang, W., Alahuhta, M., Vander Wall, T., Baker, J.O., Taylor, L.E., Decker, S.R., Himmel, M.E., and Zhang, M. (2014). Engineering towards a complete heterologous cellulase secretome in Yarrowia lipolytica reveals its potential for consolidated bioprocessing. *Biotechnology for Biofuels* 7**,** 148.

Wei, H., Wang, W., Alper, H.S., Xu, Q., Knoshaug, E.P., Van Wychen, S., Lin, C.-Y., Luo, Y., Decker, S.R., and Himmel, M.E. (2019). Ameliorating the metabolic burden of the co-expression of secreted fungal cellulases in a high lipid-accumulating Yarrowia lipolytica strain by medium C/N ratio and a chemical chaperone. *Frontiers in microbiology* 9**,** 3276.

Xu, Q., Knoshaug, E.P., Wang, W., Alahuhta, M., Baker, J.O., Yang, S., Wall, T., Decker, S.R., Himmel, M.E., Zhang, M., and Wei, H. (2017). Expression and secretion of fungal endoglucanase II and chimeric cellobiohydrolase I in the oleaginous yeast Lipomyces starkeyi. *Microbial cell factories* 16**,** 126.
